# Retinomorphic Visual Processing Enabled by Contact‐Engineered IGZO Optoelectronic Synaptic Memtransistors

**DOI:** 10.1002/advs.76218

**Published:** 2026-06-25

**Authors:** Donghyun Kang, San Nam, Dayul Nam, In‐Soo Kim, Myung‐Gil Kim, Sung Kyu Park, Yong‐Hoon Kim

**Affiliations:** ^1^ School of Advanced Materials Science and Engineering Sungkyunkwan University Suwon South Korea; ^2^ School of Electrical and Electronics Engineering Chung‐Ang University Seoul South Korea; ^3^ Department of Medicine University of Connecticut Health Center Farmington Connecticut USA

**Keywords:** contact engineering, indium–gallium–zinc–oxide, memtransistors, optoelectronic, visual processing

## Abstract

Driven by the rapid progress of artificial intelligence and robotics, neuromorphic vision systems are gaining significant attention for enabling efficient visual information processing in complex and dynamic environments. In particular, optoelectronic devices that emulate the functionality of the biological retina are essential for achieving efficient neuromorphic visual processing. Here, we report an optoelectronic synaptic memtransistor (OSMT)‐based neuromorphic vision system for image processing applications. By integrating photoresponsive indium–gallium–zinc–oxide (IGZO) as the channel material with a hafnium oxide (HfO_2_) contact‐engineered architecture, the OSMT exhibits optically and electrically tunable resistive states, enabling stable and controllable synaptic weight modulation for artificial neural network (ANN) implementation. Benefiting from reliable optoelectronic synaptic characteristics, ANN simulations achieve a handwritten digit recognition accuracy of 92.17%. Furthermore, a 6 × 6 OSMT array demonstrates neuromorphic image processing capabilities, including contrast enhancement. These results highlight the potential of OSMTs as key building blocks for intelligent machine vision systems, offering new opportunities for advanced robotic platforms and human–machine interfaces.

## Graphical abstract

Here, we present an optoelectronic synaptic memtransistor (OSMT) integrating photoresponsive IGZO with contact‐engineered HfO_2_, enabling electrically and optically tunable synaptic weights. The device demonstrates broad range of tunable conductance states and array‐level image processing, highlighting its potential for intelligent machine vision.

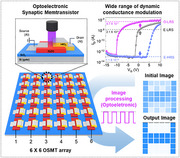

## 1 Introduction

Inspired by the remarkable capabilities of the biological visual system, neuromorphic vision systems are receiving increasing attention in the field of artificial machine vision [1]. In the human visual pathway, incident light is focused onto the retina, where it is sensed by photoreceptors and undergoes essential visual information processing [2]. These retinal processes include contrast enhancement and edge detection, enabling efficient feature extraction prior to signal transmission to higher visual centers [3]. The functional principles of retinal processing provide a direct blueprint for neuromorphic vision system design. In particular, the retina's ability to simultaneously sense optical stimuli and modulate synaptic transmission inspires in‐sensor and in‐retina computing paradigms, in which sensing and processing are intrinsically integrated. To emulate these capabilities, neuromorphic vision systems require synaptic devices that can directly respond to optical inputs while adaptively adjusting synaptic weights through electrical signals. Since synaptic weight modulation is typically represented by changes in device conductance or resistance [4], synaptic devices whose conductance can be reliably tuned by both optical and electrical stimuli are highly desirable for realizing retina‐inspired neuromorphic vision systems.

In this regard, a wide range of optoelectronic devices have been explored for neuromorphic vision systems, among which metal oxide‐based platforms have attracted particular attention due to their complementary‐metal‐oxide‐semiconductor compatibility, large‐area scalability, and robust electrical properties [5, 6, 7]. Moreover, oxide semiconductors such as indium–gallium–zinc–oxide (IGZO) exhibit persistent photoconductivity (PPC), enabling effective coupling between optical sensing and synaptic weight modulation in biomimetic vision devices [8, 9, 10]. Previously, various optoelectronic memristors based on metal oxides were demonstrated [11, 12, 13, 14]. Building upon these memristive concepts, Lin et al. reported an event‐driven retinomorphic photodiode that integrates metal‐oxide interfaces with organic heterojunctions to replicate the retina's signal pathway within a two‐terminal structure, achieving environment‐adaptive perception [15]. Nevertheless, the two‐terminal architecture of memristors might induce complex circuitry challenges when integrated into neuromorphic systems, primarily due to the presence of sneak current paths [16, 17]. To tackle this issue, three‐terminal optoelectronic synaptic transistors emerged as a reliable alternative [18, 19]. Kim et al. demonstrated the potential of transistor‐based integration by developing neuromorphic visual receptive field hardware, where IGZO optomemristors are vertically integrated over silicon transistors to emulate biological hierarchical signal processing [20]. By decoupling the signal transmission and weight modulation pathways, three‐terminal devices offer precise and wide‐range conductance tunability, intrinsic transistor functionalities for signal amplification and gating, and robust, crosstalk‐free emulation of complex synaptic behaviors [21]. Furthermore, optoelectronic memtransistors uniquely integrate nonvolatile memory characteristics with optically controlled synaptic operation, enabling persistent conductance states with exceptionally long retention times. Unlike conventional optoelectronic synaptic transistors, which typically rely on transient photoresponse, optoelectronic memtransistors can preserve synaptic weights in the absence of both electrical and optical stimuli. This intrinsic nonvolatility ensures stable long‐term memory storage and lower energy consumption, thereby broadening their applicability for memory or logic applications [22, 23, 24].

Here, we present an IGZO‐based optoelectronic synaptic memtransistor (OSMT) for neuromorphic vision systems, enabling wide‐range and multimodal synaptic modulation via combined electrical and optical stimulation. The proposed OSMT integrates electrical biasing and optical excitation within a single memtransistor architecture, enabling multi‐level conductance control with reliable retention times and rendering it intrinsically suitable for vision‐oriented neuromorphic computing. Particularly, a thin (7.5 nm) hafnium oxide (HfO_2_) interlayer asymmetrically embedded at the channel/drain interface enabled electrically driven high‐ and low‐resistance states (E‐HRS and E‐LRS). In addition, the use of light‐sensitive IGZO as the channel allowed an optically driven low‐resistance state (O‐LRS), significantly extending the synaptic modulation window beyond the electrical operation. Owing to this dual‐stimulation scheme, the OSMT successfully emulated key synaptic functions including short‐term memory/long‐term memory (STM/LTM) and paired‐pulse facilitation (PPF), and potentiation/depression behaviors. As a result, an artificial neural network (ANN)‐assisted image recognition accuracy of 92.17% was achieved. Furthermore, a retina‐inspired visual processing application is demonstrated, underscoring the advantage of wide and multimodal conductance tunability. These results highlight oxide OSMTs as a promising synaptic device platform that intrinsically unifies optical sensing and synaptic weight modulation for advanced neuromorphic vision systems.

## 2 Results and Discussion

### 2.1 Conceptual Demonstration and Optoelectronic Analysis of OSMTs

**FIGURE 1:**
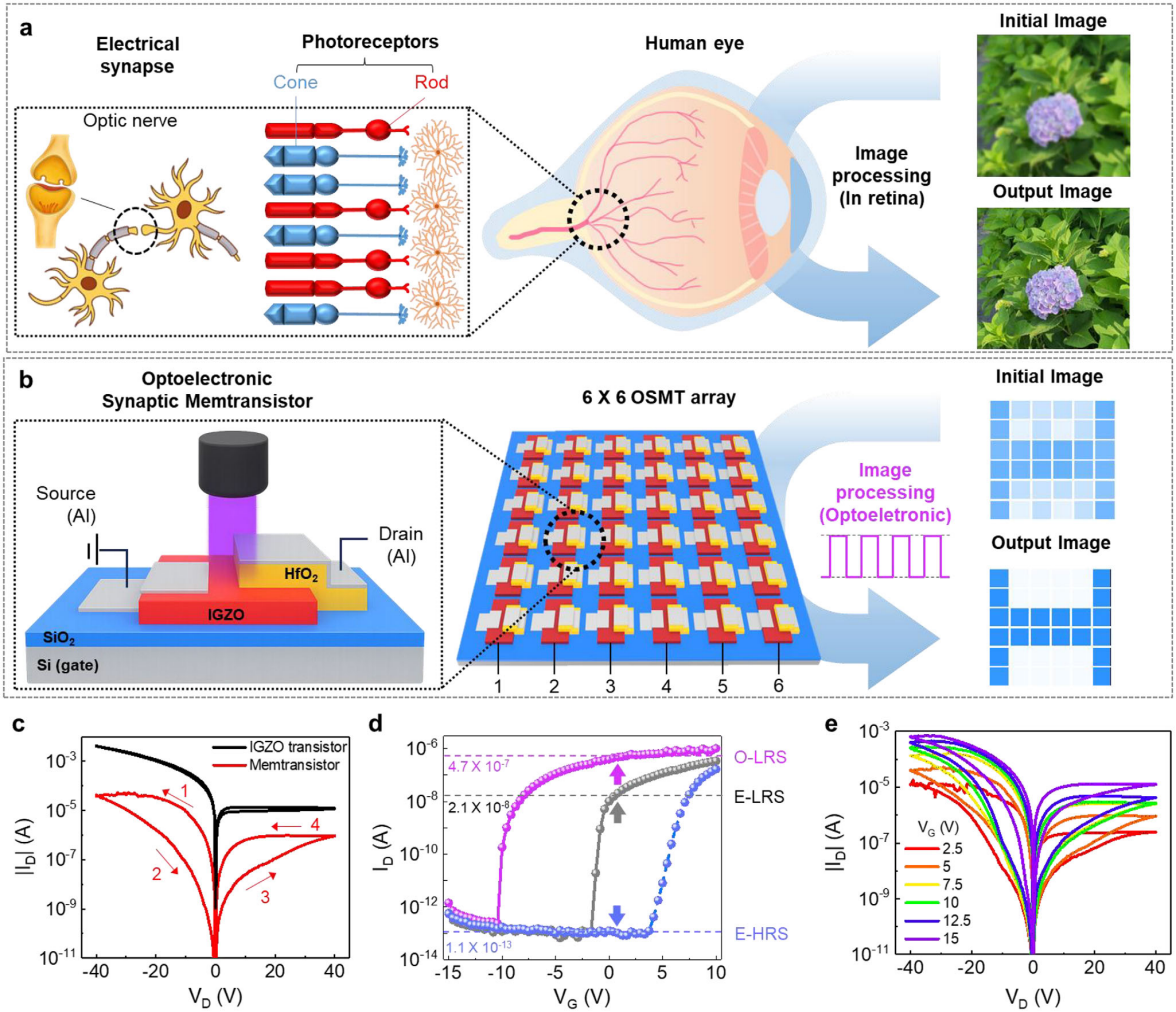
Device concept, architecture, and optoelectronic characteristics of OSMTs. (a) Schematic diagram of a human retina and the image processing functions. Input image with low contrast undergoes image processing in the retina, thus changing into a high‐contrast image. (b) Architecture of the array and the individual OSMT device. The channel width and length were 200 µm and 40 µm, respectively. Utilizing each device as an image pixel, we controlled the optical inputs for image processing. (c) Resistive switching characteristics of OSMTs on a log scale. The direction of V_D_ sweep is indicated by arrows. (d) Transfer characteristics of OSMT. The blue, black, and purple dashed lines indicate the E‐HRS, E‐LRS, and O‐LRS, respectively. (e) The gate tunable resistive switching characteristics of OSMTs.

From a biological perspective, vision is achieved through a series of complex and hierarchical processes. Especially, the retina lies at the core of human vision, functioning as a central processor that transforms the optical input into electrical output. Figure 1a depicts the structure of a human retina and the retinal preprocessing of an image. The outer edges of the retina contain two types of photoreceptors including rods and cones, which are essential for visual perception [25]. These rods and cones have light‐absorbing visual pigments that enable the detection of incident light. When the light passes through the optic nerves and reacts with the pigments, the pigments undergo a chemical change that modifies the cell membrane permeability. This change triggers early‐stage visual preprocessing including contrast enhancement, after which the processed information is transmitted to the brain through interconnected neuronal pathways via the optic nerve. To demonstrate the image processing functions of the biological retina, a 6 × 6 array based on OSMTs was implemented having a contact‐engineered structure (Figure 1b). Here, the OSMT was fabricated with a channel length of 40 µm, and a 7.5 nm‐thick HfO_2_ interlayer was placed between the IGZO channel and the aluminum (Al) drain electrode. The OSMT can mimic the features of a retina since the IGZO channel has the capability to respond to light, corresponding to the photoreceptors in the retina. By leveraging synaptic weights tunable via both optical and electrical stimuli, a 6 × 6 OSMT array was implemented as an image‐processing system for contrast enhancement. In this image‐processing architecture, each OSMT device functions as a single pixel unit, establishing a one‐to‐one correspondence between the physical device array and the image array. Thus, an initial image was mapped according to the initial conductance states of 36 OSMT devices. Akin to the biological vision system, the OSMT‐based image processing system can refine the initially mapped image utilizing an optical pulse scheme, improving feature distinction and notably enhancing the contrast of the image. The detailed contrast enhancement procedure is discussed in a later section.

Figure 1c compares the resistive switching characteristics (I_D_‐V_D_) of IGZO transistor and OSMT, plotted on a log scale of drain current (I_D_) with a fixed gate bias of +5 V. For both devices, the V_D_ was swept in the sequence of 0 V → −40 V → 0 V → +40 V → 0 V. In the case of IGZO transistor, a typical I_D_‐V_D_ characteristic without hysteresis was observed with the saturation of I_D_ at high positive V_D_ region. On the other hand, OSMT exhibited a wide hysteresis, clearly demonstrating the memory characteristics of memtransistors. In sweep 1, the I_D_ was increased up to ∼10^−4^ A with the increase of negative V_D_, indicating the initial E‐LRS. During the sweep 2, the I_D_ level decreased creating a wide hysteresis, showing the transition from E‐LRS to E‐HRS. Afterward, the device was gradually set to E‐LRS during the sweep 3. In the final sweep 4, the device maintains E‐LRS, showing a high I_D_ level of ∼10^−6^ A. To explain the resistive switching phenomenon, transfer characteristics (I_D_‐V_G_) of OSMTs were systematically analyzed. The transfer curves were obtained for the E‐HRS, E‐LRS, and O‐LRS, which were programmed by applying a V_D_ of −40 V, a V_D_ of +40 V, and UV illumination, respectively. As shown in Figure 1d, a pronounced shift in the turn‐on voltage (V_on_) is observed, clearly differentiating the E‐HRS, E‐LRS, and O‐LRS. In the E‐HRS, positive shift of V_on_ was observed (V_on_ ∼ 3.5 V). After applying V_D_ of +40 V, E‐LRS was obtained with a negative shift of V_on_ (V_on_ ∼ −2 V). Upon further application of UV light, V_on_ shifted to ∼ −11 V. When the OSMT is illuminated with UV light pulses, photoelectrons are generated within the IGZO channel via band‐to‐band excitation and the ionization of oxygen vacancies [26], contributing to the negative shift of transfer characteristics. As a result, at V_G_ of 1.25 V (indicated by the arrow), 3 distinct resistive states can be attained: E‐HRS, E‐LRS, and O‐LRS, with the current ratio of O‐LRS to E‐HRS reaching up to ∼10^6^. The evolution of I_D_‐V_G_ curves with respect to the transition from O‐LRS to E‐HRS is further presented in Figure S1 with more detail. The I_D_‐V_D_ characteristic under UV illumination is also shown in Figure S2, showing elevated conductance states at V_G_ = 0 V. These results clearly demonstrate the formation of a memory window and the wide conductance tunability of OSMTs. While designing the OSMT with a downscaled channel length of 20 µm leads to a decrease in the memory window, it effectively allows for a reduction in the required sweep voltage ranges (Figure S3). Additionally, Figure 1e shows the gate‐tunable memristive characteristics of OSMTs. Through gate‐bias modulation, broader range of conductance states can be acquired, which is a crucial feature that differentiates the three‐terminal memtransistors from two‐terminal synaptic devices. Here, the V_G_ was increased from 2.5 V to 15 V with a 2.5 V step, and the on‐state current (I_LRS_) increased from ∼10^−7^ A to ∼10^−5^ A. This pronounced gate‐controlled current modulation demonstrates that synaptic conductance in OSMTs can be synergistically tuned by both gate and drain biases. Such electrical control enables an expanded memory window and a finer granularity of synaptic weight states, which are critical for advanced neuromorphic computing and vision systems.

### 2.2 Memory Characteristics and Resistive Switching Mechanism of Memtransistors

**FIGURE 2:**
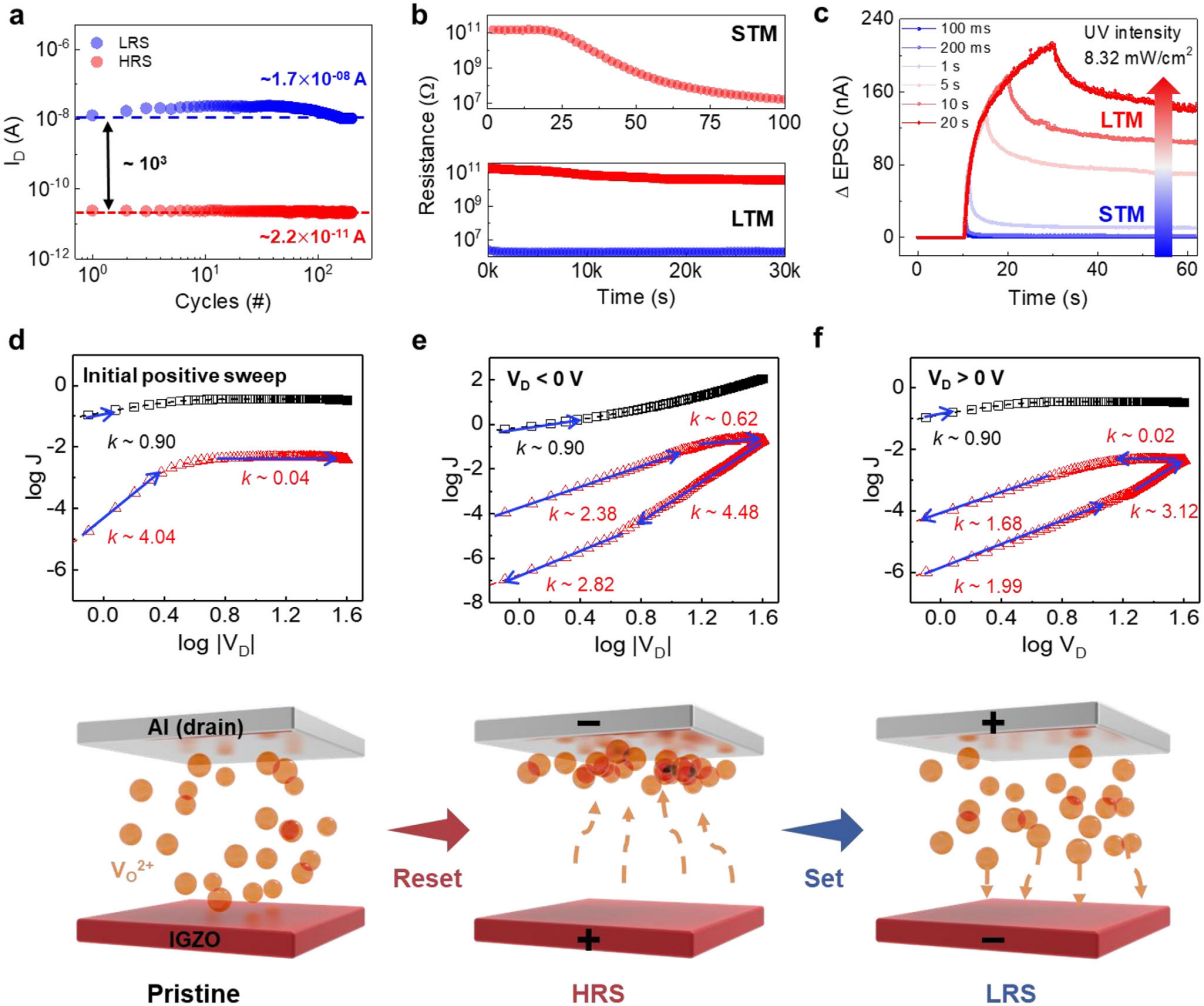
Optoelectronic memory characteristics and the resistive switching mechanism of OSMTs. (a) Endurance characteristics of OSMTs at LRS and HRS for 200 consecutive sweep cycles (V_G_ = 5 V, V_D_ = 5 V). (b) Retention behavior emulating the STM and LTM states. The I_D_ current was read at V_G_ = 5 V, V_D_ = 5 V. (c) ΔEPSC triggered by UV input with fixed intensity, but different input times. Short and long optical input induces STM and LTM behaviors, respectively. J‐V characteristics of OSMT and the schematics of oxygen vacancy migration in HfO_2_ interlayer during the (d) initial positive sweep, (e) negative V_D_ region, and (f) positive V_D_ region. The black and red line represents IGZO transistor and OSMT, respectively.

To evaluate the memory characteristics of OSMTs, diverse electrical and optoelectronic measurements were carried out. Figure 2a shows the cycling endurance characteristics of OSMTs. To program the device into the E‐LRS, a positive voltage pulse of +40 V with a duration of 500 ms was applied, whereas a negative voltage pulse of −40 V and identical width was employed to induce the E‐HRS. High I_LRS_ of ∼10^−8^ A and low off‐state current of ∼10^−11^ A were continuously acquired at V_G_ = 5 V and V_D_ = 5 V for more than 10^2^ cycles, demonstrating stable memristive properties. While the proposed OSMT architecture provides a robust platform for implementing key retinomorphic functions discussed later in this work, the current endurance characteristics might present a limitation for immediate practical hardware integration. The degradation in endurance is attributed to the diffusion of oxygen vacancies within the HfO_2_ switching layer under continuous stress and the subsequent annihilation of oxygen vacancies during repeated switching cycles. To mitigate this defect‐driven degradation and secure enhanced operational stability, we adjusted the electrical bias conditions for endurance measurement, specifically by increasing the voltage sweep range to reduce the out‐diffusion and annihilation of oxygen vacancies. As a result of this tailored bias engineering, the device achieved an improved endurance profile, successfully extending its stable operational window up to 300∼400 cycles without catastrophic readout failure (Figure S4). While further material‐level refinements remain a focus of our ongoing work, this successful bias‐induced stabilization firmly demonstrates that the reliability bottleneck of our OSMT system can be effectively managed through strategic operational tuning, bolstering its viability for practical neuromorphic hardware implementations. Furthermore, with the OSMT, dynamic transition between the STM and LTM states can be achieved by modulating the conditions of electrical inputs. Figure 2b illustrates the short‐term and long‐term retention states controlled by the duration of electrical input pulses, where the blue and red lines indicate the E‐LRS and E‐HRS retention curves, respectively. In the case of STM (V_D_ = −40 V, 5 s), high resistance of ∼10^11^ Ω was only maintained for approximately 25 s and rapidly decayed down to ∼10^7^ Ω (upper panel of Figure 2b). The use of shorter electrical inputs (1 and 3 s) resulted in a rapid E‐HRS to E‐LRS transition, suggesting that retention time is critically governed by the temporal length of the input stimulus (Figure S5). On the other hand, with longer negative electrical input (V_D_ = −40 V, 12 s), E‐HRS was maintained for ∼30 ks (resistance of 10^10^ ∼ 10^11^ Ω), exhibiting LTM behavior. Under positive electrical input (V_D_ = +40 V, 2 s), the E‐LRS could be also maintained up to 30 ks, demonstrating reliable long‐term retention properties. Moreover, Figure 2c shows the STM‐to‐LTM transition enabled by modulating the optical input duration. Here, UV light was exposed with a fixed intensity of 8.32 mW∙cm^−2^ with the duration varying from 100 ms to 20 s. With shorter durations (0.1 ∼ 1 s), STM behavior was observed, with a negligible increase of excitatory postsynaptic current (EPSC). However, when the input duration exceeded 1 s, transition from STM to LTM occurred. With the longest exposure of 20 s, the retention current increased up to 160 nA, where the retention current is defined as the current measured 30 s after the light exposure (Figure S6). Controlling the PPC behavior of oxide semiconductors is a widely utilized strategy to achieve tunable memory states, where the PPC behavior is induced from the gradual neutralization of ionized oxygen vacancies after the removal of light illumination [27].

Conventionally, resistive switching mechanisms of oxide‐based memory devices can be divided into filament‐type conduction and gradual‐type conduction [28, 29]. However, in our device, filament‐type mechanism is not applicable since no forming process or abrupt increase of device current was observed. Therefore, we propose a gradual‐type resistive switching mechanism based on the migration of oxygen vacancies, where the vacancies serve as direct electron conducting paths. To describe the resistive switching mechanism, the power‐law behaviors (J∝V^k^) in OSMTs and IGZO transistors with Al electrode were plotted on a double‐logarithmic scale (Figure 2d–f). Schematic diagrams of the resistive switching process are also presented based on the field‐induced migration of oxygen vacancies. Here, J is the current density and k is the slope of the linear region in the J‐V curve. The J‐V characteristics were extracted from the I_D−_V_D_ curves in Figure 1c. To compare the initial state of OSMTs and IGZO transistors, a positive V_D_ sweep was carried out for as‐fabricated devices (Figure 2d). For IGZO transistors (black squares), an immediate increase in I_D_ with Ohmic conduction behavior (k ≈ 1) was observed, indicating the formation of an ideal Ohmic contact at the channel/electrode interface. In contrast, OSMTs displayed a more gradual increase in I_D_, suggesting a higher contact resistance at the channel/electrode interface arising from the insertion of the HfO_2_ interlayer. The distinct I_D−_V_D_ characteristics observed in the low V_D_ regime for the two device types are consistent with the proposed operating mechanism (Figure S7). Nevertheless, the initial state of the OSMTs can be regarded as E‐LRS, owing to their forming‐free behavior and relatively high initial current density of approximately 10^−5^ A∙cm^−2^. The initial LRS can be explained by the uniformly distributed oxygen vacancies (orange spheres denoted as V_O_^2+^ in Figure 2d–f) in the pristine HfO_2_ interlayer, since the oxygen vacancies can serve as electron conducting paths [30]. For further experimental verification, I‐V characteristics were measured from the as‐fabricated IGZO diode based on an Indium tin oxide/IGZO/HfO_2_/Al vertical structure (Figure S8). The results clearly demonstrate that the pristine HfO_2_ layer does not exhibit ideal insulating behavior but instead shows appreciable conductivity. Moreover, we systematically examined the effect of geometric scaling on the resistive switching behavior, since area‐dependent current scaling behaviors provide solid evidence for a typical gradual‐type switching mechanism. As shown in Figure S9, the device demonstrates a clear dependence of switching behavior on the interlayer area, with larger areas yielding a marked increase in electrical conductivity. Specifically, we analyzed the relationship between the switching area and I_LRS_ which is defined as the maximum E‐LRS current. The result displays an approximately linear correlation between the I_LRS_ and the switching area, suggesting that the overall conduction is not governed by a single conductive filament, but rather reflects a distribution of defect sites acting as conduction pathways. Furthermore, we observed the linear dependence between the switching area and the set voltage, which is defined as the voltage point where the transition from E‐HRS to E‐LRS is initiated. This proportional relationship can be attributed to the intrinsic properties of the HfO_2_ switching layer where a larger physical area inherently accommodates a greater population of oxygen vacancies and defect clusters. Such an increased defect density enhances the probability of forming a more spatially extended conduction pathways under an applied electric field. In the negative V_D_ sweep condition (Figure 2e), IGZO transistors once again exhibit Ohmic behavior (k ≈ 1) although with higher current density compared to the positive V_D_ sweep condition. On the contrary, OSMTs exhibit a pronounced hysteresis loop in their current–voltage characteristics. The current density rapidly increases from ∼10^−4^ A∙ cm^−2^ with the slope of 2.38 at V_D_ below −10 V. At higher voltages, the exponent k reduces to ∼ 0.62. For the backward sweep, the current density abruptly decreases from ∼10^−1^ A∙cm^−2^ to ∼10^−7^ A∙cm^−2^, creating hysteresis. Under this bias condition, positively charged oxygen vacancies are expected to drift toward the drain electrode in accordance with the applied electric field, leading to the observed memristive behavior. Here, the electric field by the gate and drain bias are same in direction, together making a strong electric field (Figure S10). Therefore, the vacancies are strongly accumulated near the drain electrode, resulting in the transition from LRS to HRS. In the subsequent positive V_D_ sweep, the current density exhibited power–law dependence (k ≈ 2) on the positive V_D_ under 10 V (Figure 2f). With further increase of voltage, the current density rises faster (k ≈ 3.12). In this case, the direction of electric field is expected to trigger the vacancies to drift downward, restoring the LRS. However, the electric field induced by the V_G_ partially counteracts the electric field generated by the drain voltage, thereby reducing the effective driving force on the vacancies. As a result, the vacancies do not strongly accumulate at the bottom interface. Consequently, the device maintains the LRS during the backward sweep.

### 2.3 Emulation of Synaptic Functions Using OSMTs

**FIGURE 3:**
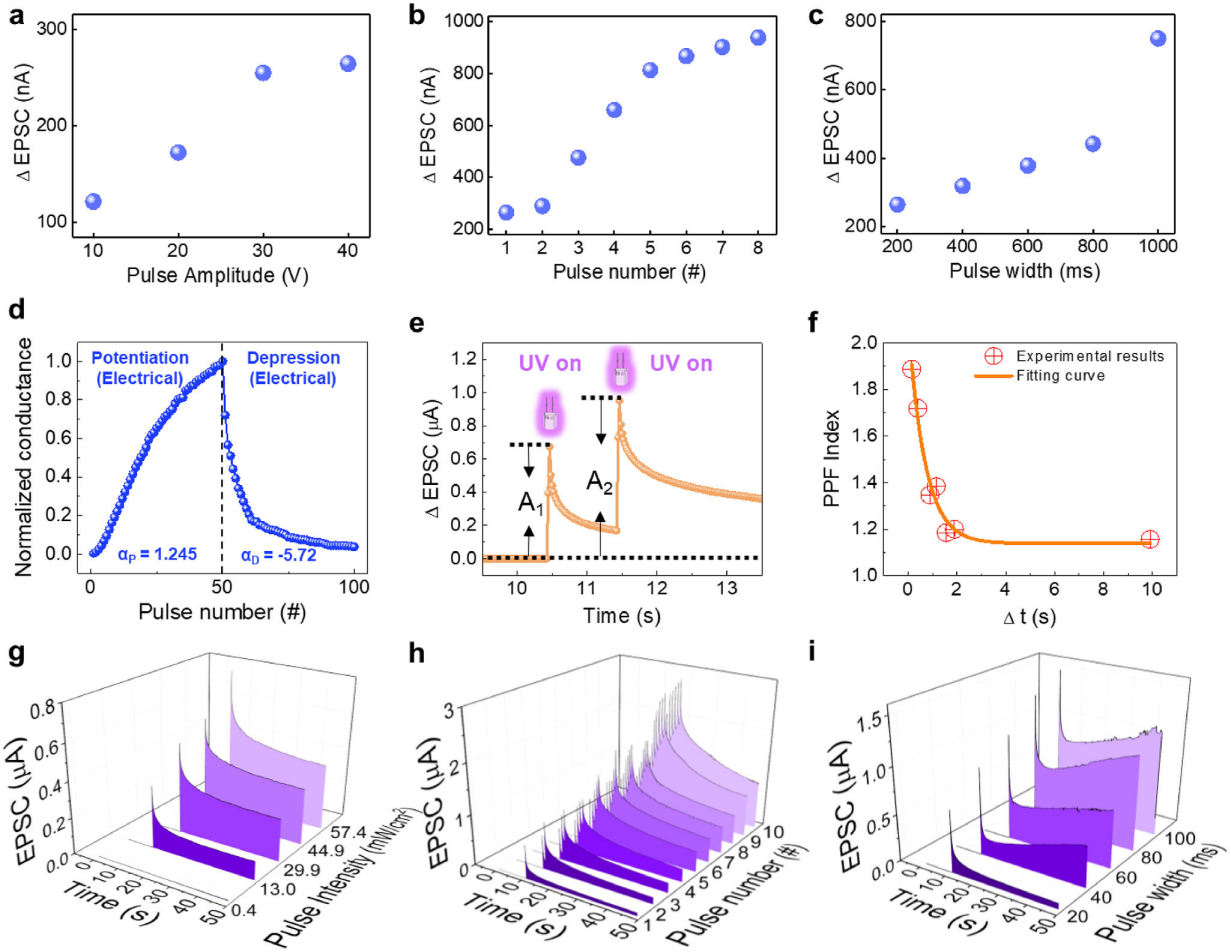
Emulation of diverse synaptic functions utilizing optoelectronic stimuli. EPSC variation as a function of electrical (a) pulse amplitude, (b) pulse number, and (c) pulse width. To measure the change in EPSC, a read pulse (pulse amplitude = 5 V, pulse width = 20 ms) was applied immediately following each electrical stimulation. (d) Emulation of LTP and LTD based on electrical‐only pulse scheme. (e) PPF characteristics of OSMTs using UV pulse. The interval was set to 1 s. (f) PPF index variation as a function of Δ*t*. EPSC variation as a function of optical (g) pulse amplitude, (h) pulse number, and (i) pulse width. The initial pulse was applied 10 s after starting the sampling process, and every measurement was conducted with fixed V_G_ and V_D_ conditions of 3 V and 5 V, respectively.

To evaluate the emulation of synaptic plasticity in OSMTs, the variations in post‐synaptic responses triggered by optoelectronic signals were analyzed. Figure 3a–c shows the changes in EPSC under varying electrical pulse conditions including pulse amplitude, number, and width. In neuronal perspective, elevated pulse amplitudes promote increased neurotransmitter release, thereby strengthening the post‐synaptic response [31]. For amplitude‐dependent measurements, pulses of 10–40 V were applied in 10 V increments with a fixed width of 200 ms (Figure 3a). The linear dependence between pulse amplitude and ΔEPSC clearly indicate a stepwise conductance modulation, showcasing the applicability of OSMTs for synaptic devices. The effect of pulse number on ΔEPSC was subsequently examined by applying 1–8 consecutive pulses (pulse amplitude = 40 V, pulse width = 200 ms). In human synapse, the repetition of electrical spikes can strengthen the synaptic connection, leading to potentiation behavior [32]. As shown in Figure 3b, analogous to the biological synapse, ΔEPSC gradually increased with the increase of pulse number, where the EPSC level reached 900 nA after 8 consecutive stimulations. In addition, the influence of pulse width was investigated by varying the width from 200 to 1000 ms. The pulse width‐dependency is akin to what happens in a biological synapse where longer spike allows more transmission of signals between neurons, inducing the solidification of synaptic strength [33]. As shown in Figure 3c, a clear dependence of ΔEPSC on pulse duration was observed.

By analyzing the above synaptic responses, we emulated the potentiation and depression behavior of human nervous system. Figure 3d shows the evolution of normalized conductance during electrically induced long‐term potentiation (LTP) and depression (LTD) processes with fixed V_G_ of 5 V. Here, for electrical potentiation, 50 consecutive pulses were applied with a V_D_ pulse width of 20 ms and an amplitude of 40 V. For electrical depression, 50 continuous pulses were applied with the same pulse width (20 ms) and an amplitude of −25 V. The voltage‐dependent LTP and LTD behaviors with fixed pulse width of 200 ms are also presented in Figure S11. During the potentiation process, a linear increasing trend of conductance was observed (nonlinearity = 1.245). On the contrary, the conductance decreasing trend of depression process was relatively nonlinear compared to the potentiation process (nonlinearity = −5.72). We acquired the nonlinearity values for potentiation and depression from the equations below [34],

(1) $$G_{\mathrm{LTP}}=\;B\left(1-e^{\left(-\frac{P}{A}\right)}\right)+G_{\min}$$

(2) $$G_{\mathrm{LTD}}=-B\left(1-e^{\left(-\frac{P-P_{\max}}{A}\right)}\right)+G_{\max}$$

(3) $$B\;=\left(G_{\max}-G_{\min}\right)\;/\left(1-e^{\left(\frac{-P_{\max}}{A}\right)}\right)$$

where G_LTP_, G_LTD_, G_min_ and G_max_ indicate the potentiation conductance, depression conductance, minimum conductance, and maximum conductance, respectively. Moreover, P, P_max_, A and B represent the pulse number, the maximum pulse number, a parameter representing the nonlinearity and a fitting constant for normalizing the range of conductance, respectively. For further assessment of repeatability and endurance of the OSMT's LTP and LTD behaviors, iterative excitation and inhibition pulse schemes were applied with fixed pulse width of 200 ms, demonstrating reliable reproducibility of synaptic functions (Figure S12).

PPF is also an important neuro‐biological phenomenon where the subsequent synaptic behavior is enhanced by the preceding pulse [35]. In synaptic devices, PPF can be represented as the PPF index which is defined as the ratio between paired postsynaptic current, with the second current being greater than the first. PPF index can be obtained from the following equation,

(4) $$PPF\;index=\frac{A_{2}}{A_{1}}$$

where A_1_ and A_2_ are the postsynaptic currents evoked by the first and second optical pulses (pulse width = 20 ms, UV intensity = 29.9 mW∙cm^−2^), respectively (Figure 3e). Figure 3f depicts the evolution of the PPF index as a function of interval time Δ*t*. Here, the PPF index curve was fitted using the double exponential decay function presented below [36].

(5) $$PPF\;index=C_{1}\cdot exp\left(-\frac{\Delta t}{\tau_{1}}\right)+C_{2}\cdot exp\left(-\frac{\Delta t}{\tau_{2}}\right)+C_{0}$$

where C_1_ and C_2_ correspond to the facilitation magnitude, and *τ*_1_ and *τ*_2_ indicate the relaxation time of the exponential function. At the shortest interval (Δ*t* = 0.15 s), the PPF index reached ∼1.89, whereas increasing Δ*t* to 9.9 s resulted in a reduced index of ∼1.15, demonstrating an inversely proportional relationship between pulse interval and synaptic facilitation. This clearly indicates that the synaptic strength of OSMTs can be modulated by the temporal spacing of input signals. Beyond temporal modulation, the synaptic weight of OSMTs can also be dynamically tuned by adjusting optical pulse intensity, number, and width (Figure 3g–i). As shown in Figure 3g, larger pulse amplitude induced larger ΔEPSC, since UV pulse with larger amplitude generates more photocarriers inside IGZO channel. Here, the pulse intensity was increased from 0.4 mW∙cm^−2^ to 57.4 mW∙cm^−2^, with fixed base V_G_ = 3 V and V_D_ = 5 V. When the pulse amplitude was 0.4 mW∙cm^−2^, a negligible synaptic response was observed. As the pulse amplitude increased up to 57.4 mW∙cm^−2^, the EPSC reached 0.8 µA, reflecting the higher generation of photocarriers in the IGZO channel at larger amplitudes. Similarly, varying the pulse number from 1 to 10 (intensity = 29.9 mW∙cm^−2^, pulse width = 20 ms) produced a near‐linear increase in EPSC, reaching ∼3 µA after 10 consecutive pulses. Finally, we investigated the effect of pulse width on ΔEPSC. Since longer pulse width allows more time for the UV light to generate photocarriers in the channel, ΔEPSC increased in proportion to the pulse width. The retention currents of Figure 3g–i are also plotted in Figure S13, confirming a clear proportional dependence of ΔEPSC on various pulse parameters. In addition to UV illumination, wavelength‐dependent synaptic characteristics were further investigated using red (635 nm), green (521 nm), and blue (450 nm) light sources (Figure S14). Quantitative extraction of ΔEPSC under 10 identical optical pulses revealed a clear wavelength dependent synaptic responses, where blue illumination produced the largest ΔEPSC, followed by green, while red illumination resulted in a pA‐level response (Figure S15). This trend is attributed to the stronger optical absorption and higher photocarrier generation efficiency at shorter wavelengths, while the limited photon energy of red light leads to insufficient carrier generation in the IGZO channel. Nevertheless, this limitation can be alleviated by optimizing the sputtering conditions of the IGZO channel, which could enhance sub‐bandgap absorption and enable a more pronounced photoresponse in the red spectral region [37]. Overall, these results indicate that the synaptic functions of OSMTs can be dynamically tuned via both electrical and optical inputs, demonstrating their potential as highly tunable optoelectronic neuromorphic devices. Furthermore, the energy consumption per synaptic weight update was calculated using the following equation [38],

(6) $$E=I_{peak}\;\cdot V_{DS}\cdot t$$

where *I*_peak_ represents the maximum current of a single pulse, *V*_DS_ is the drain voltage, and *t* is the pulse width. Under the pulse condition of *V*_DS_ = 5 V and *t* = 20 ms with a measured *I*_peak_ of 0.7 µA, the energy consumption was approximately 70 nJ/pulse, demonstrating a competitive energy efficiency.

### 2.4 Emulation of LTP/LTD and ANN Performance

**FIGURE 4:**
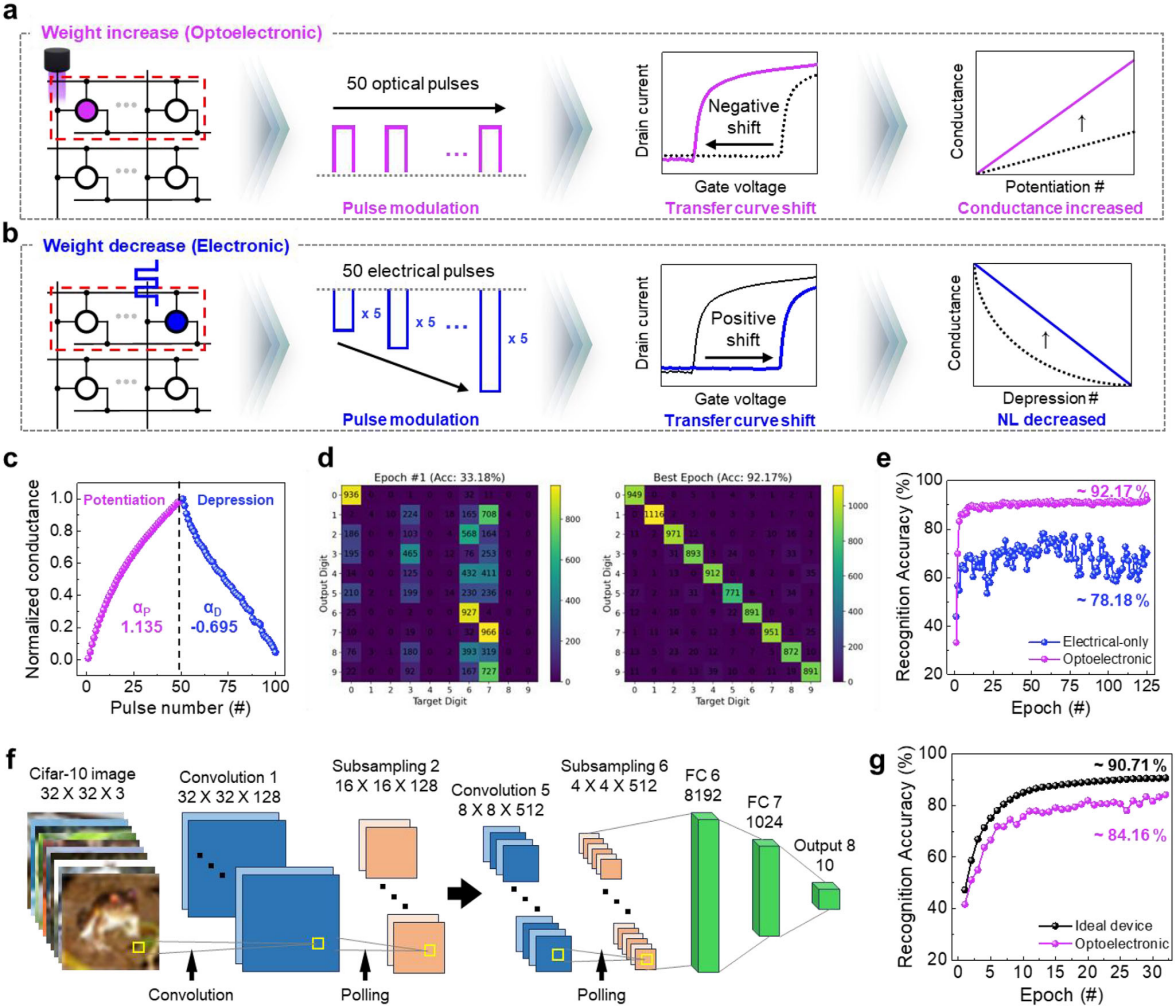
Emulation of LTP and LTD processes followed by ANN‐based digit image recognition test. (a) Schematic of the optical weight increasing process for LTP. 50 identical optical pulses induce a substantial negative shift of transfer characteristics, leading to a further increase in device conductance. (b) Illustration of the electrical weight decreasing process for LTD. 50 electrical pulses with a stepwise decrease in the amplitude trigger a gradual positive shift of transfer characteristics, leading to an enhanced nonlinearity value. (c) Evolution of normalized conductance as a function of pulse number, showing the LTP and LTD. The pulse train consists of 50 consecutive optical pulses followed by 50 consecutive electrical pulses. (d) Confusion matrices of the classification results at the epoch #1 (lowest accuracy) and best epoch (highest accuracy). (e) Handwritten digit image recognition accuracy as a function of training epochs based on different input signals. (f) Schematic of the CNN architecture designed for CIFAR‐10 image recognition. (g) Recognition accuracy for the CIFAR‐10 dataset as a function of training epochs.

High linearity and a wide synaptic weight update range are critical prerequisites for the practical implementation of synaptic devices in high‐performance neuromorphic computing systems [39, 40]. While the OSMT exhibits notable nonlinearity during potentiation (Figure 3d), high nonlinearity is observed in the depression process under electrical stimulation, posing a key challenge. To address this issue, an optoelectronic pulse modulation scheme was carried out. Figure 4a illustrates the weight update process for LTP utilizing UV optical stimuli. Here, 50 optical pulses were applied to induce the synaptic weight increase. Upon UV‐irradiation, photoelectrons are generated within the IGZO, inducing a negative shift in the transfer characteristics. Notably, the optical modulation resulted in a steeper and larger increase in conductance compared to the electrical‐only stimulation. This enhanced conductance update ratio allows for a wider dynamic range of synaptic weights essential for increasing the learning efficiency of neuromorphic computing. Conversely, the LTD characteristics were emulated utilizing electrical pulses, as illustrated in Figure 4b. Although prior studies have reported LTD with constant electrical stimulation, these approaches may be prone to conductance saturation and high nonlinearity, which can degrade overall learning performance [41]. To mitigate this issue and enhance the linearity, a stepwise pulse amplitude increment scheme was adopted for the depression process. In this scheme, the magnitude of the negative V_D_ was incrementally increased every 5 pulses within the 50‐pulse train. Upon applying these electrical pulses, a positive shift in the transfer curve was observed, which is attributed to the field‐induced migration of oxygen vacancies depicted in Figure 2e. Unlike the constant pulse method utilized during LTD process in Figure 3d, the stepwise increase in V_D_ provided a more linear conductance update, effectively compensating for the saturation of conductance change as the device transitions to E‐HRS. As a result, the nonlinearity value was significantly decreased, enabling a more linear and symmetric synaptic weight update.

Figure 4c presents the normalized conductance evolution during LTP and LTD, achieved using the combined scheme of 50 optical pulses for LTP and 50 amplitude‐modulated electrical pulses for LTD. For the optical weight increase process, 50 identical UV optical pulses (pulse width = 20 ms, intensity = 29.9 mW∙cm^−2^) were applied and the response was read with fixed V_G_ = 5 V and V_D_ = 3 V conditions. For LTD process, a total of 50 pulses that consist of 10 sets of amplitude‐modulated pulses were utilized. Specifically, the pulse train started with −7 V and the applied voltage decreased with −2 V step. Five pulses were delivered at each voltage level, constituting one set. This procedure was repeated ten times, corresponding to voltage levels of −7, −9, −11 V, and so forth. Consequently, a total of 50 electrical pulses were applied, with the final pulse amplitude reaching −25 V. From the acquired LTP and LTD characteristics, we extracted the nonlinearity values. Compared to the device in Figure 3d, the device exhibited highly linear potentiation (α_P_ = 1.135) and depression (α_D_ = −0.695) characteristics. Based on these obtained synaptic characteristics, the recognition of Modified National Institute of Standards and Technology (MNIST) handwritten digit images was simulated to evaluate the feasibility of the ANN system. A fully connected multilayer perceptron ANN comprising an input layer (400 input neurons), a hidden layer, and an output layer (10 output neurons) was utilized (Figure S16). The 400 inputs correspond to the 20 times 20 pixels of the MNIST image, while the 10 outputs represent the digits 0–9. The simulation was conducted by training with images randomly selected from the 60,000 training dataset and evaluated using the 10,000 testing dataset. Figure 4d shows the confusion matrices obtained at the initial epoch (epoch #1) and the epoch corresponding to the highest recognition accuracy. While the matrix of initial epoch shows random classification with low accuracy of 33.18%, the best epoch demonstrates clear diagonal dominance with the overall accuracy of 92.17%, indicating successful learning and classification of the input patterns. For comparison, confusion matrices derived from the data in Figure 3d are provided in Figure S17, further highlighting the enhanced ANN performance of OSMTs under the optoelectronic pulsing scheme. Figure 4e shows the evolution of recognition accuracy as a function of training epochs for electrical‐only and optoelectronic LTP/LTD processes, respectively. While the electrical modulation scheme reaches a saturated accuracy of ∼78.18%, the optoelectronic pulse modulation achieves a markedly higher accuracy of 92.17%. This substantial improvement demonstrates that optoelectronic operation enables high linearity and a wide dynamic range of synaptic weights, which are essential for maximizing learning efficiency and classification performance in neuromorphic computing systems.

In addition to the MNIST benchmark, we implemented a Convolutional Neural Network (CNN) simulation, a more mainstream neural network architecture, to further assess the device's applicability to actual visual systems. As illustrated in Figure 4f, the implemented CNN was evaluated using the CIFAR‐10 dataset, which consists of high‐dimensional color images with varied backgrounds and textures, providing a more rigorous benchmark for realistic visual recognition tasks [42]. This architecture incorporates biologically inspired hierarchical processing mechanisms, where convolutional layers perform effective feature extraction by mimicking the receptive fields of the human visual cortex, while subsampling (pooling) layers introduce spatial invariance to ensure robust recognition regardless of positional variations within the input frame. By mapping the experimentally obtained optoelectronic LTP/LTD characteristics into the CNN synaptic weights, the classification performance was evaluated. As shown in Figure 4g, the system achieved a high recognition accuracy of 84.16% for the CIFAR‐10 dataset under the optoelectronic pulse modulation scheme, which closely approaches the 90.71% accuracy achieved by a simulation utilizing an ideal synaptic device. In contrast, the simulation using the electrical‐only LTP/LTD process failed to demonstrate significant training progress for the CIFAR‐10 dataset, primarily due to the excessively high nonlinearity during the depression process. These results underscore that our proposed memtransistor is not limited to simple pattern recognition but possesses substantial potential for mimicking complex visual systems and effectively processing sophisticated optical information beyond simple MLP models.

### 2.5 Image Processing System Based on a 6 × 6 OSMT Array

**FIGURE 5:**
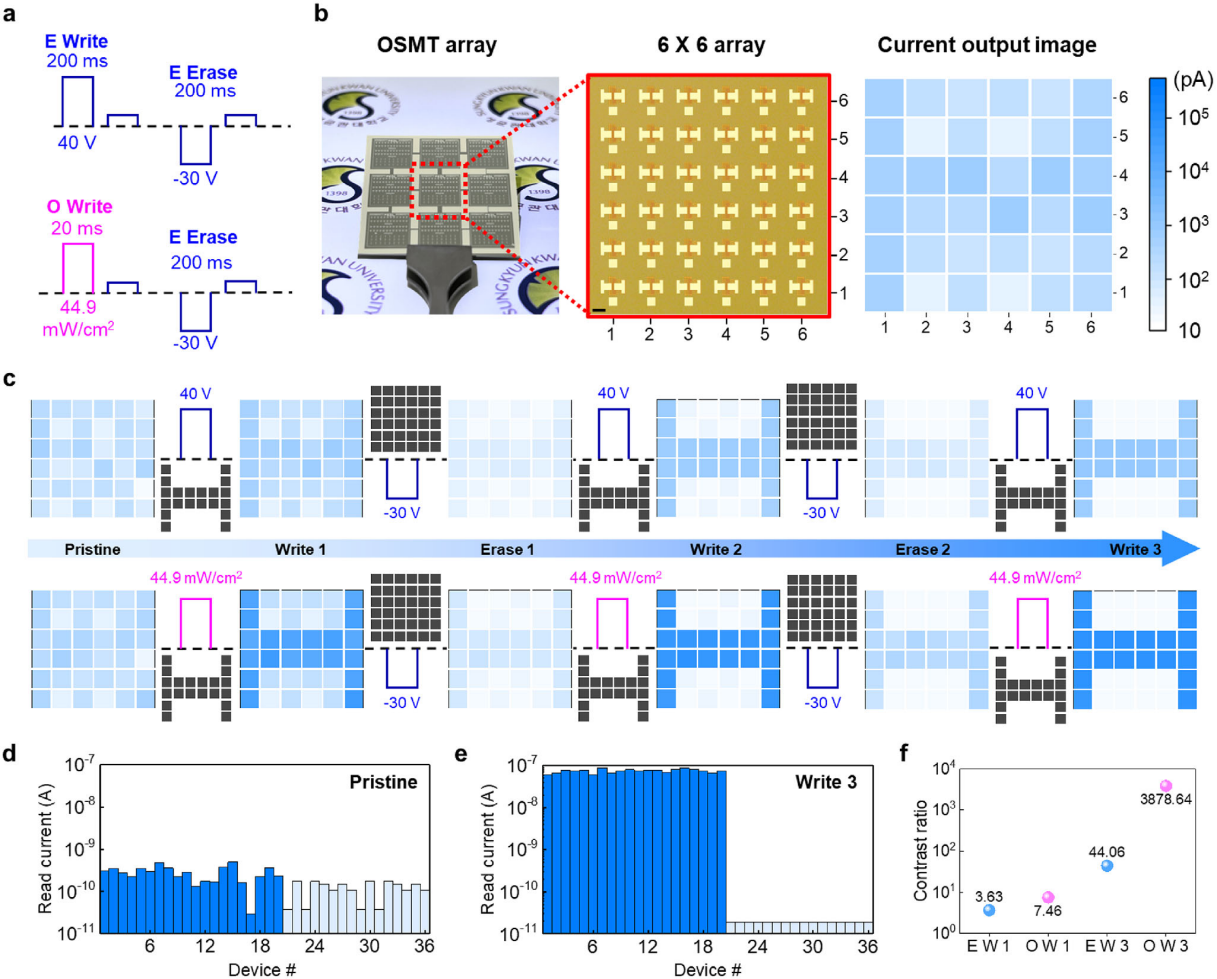
Demonstration of OSMT array for image processing. (a) Diagram of pulsing scheme for image processing. (b) Optical microscope image of a 6 × 6 OSMT array and the image mapping result after the initial write process. The scale bar indicates 100 µm. The 36 OSMT devices represent individual pixels in the image array. (c) Demonstration of image processing task. The evolution of images indicates the enhancement of image contrast, showing a clear blue letter “H.” The comparison of current values between 20 pixels representing letter “H” and 16 background pixels after d) initial optical write process, and e) final optical write process. f) The initial and final contrast ratio of the electrically and optically stimulated array image. “EW” and “OW” represents electrical and optical write, respectively.

Exploiting the widely tunable resistive states of OSMTs, we fabricated a 6 × 6 device array that integrates optical sensing and synaptic weight modulation, thereby enabling a retina‐inspired image processing platform. Here, a certain set of electrical and optical pulses was applied to the OSMT array to generate an image and enhance the contrast of the generated image. Figure 5a depicts a diagram of the pulse scheme applied to the OSMT array. For both types of stimuli, pulses were applied in a repeated sequence of write‐read‐erase‐read. In electrical image processing, write and erase pulses of identical width (200 ms) were utilized, with different amplitudes of 40 V and −30 V, respectively. For optical image processing, UV pulse (width = 20 ms, intensity = 44.9 mW∙cm^−2^) was used as the write pulse, whereas the erase pulses were identical to those used in electrical processing. The read pulses were globally fixed as V_G_ = 5 V and V_D_ = 3 V. Figure 5b shows the structure and optical microscopy image of the 6 × 6 OSMT array, and current mapping data measured from the array. In this configuration, each OSMT device corresponds to a single pixel in the image mapping process. We converted the initial currents of OSMT devices read in V_D_ = 3 V into pixel values, where higher current value represents blue color. To ensure the reliability of the array‐integrated system, we evaluated the device‐to‐device uniformity of the 6×6 OSMT array, which exhibited a highly consistent resistance state distribution at a fixed V_G_ of 10 V (Figures S18–S19). This spatial uniformity was further evidenced by the statistical analysis of core synaptic responses, such as EPSC and LTP/LTD, conducted across all 36 devices. The detailed statistical evaluation, including the mean and standard deviation values for these synaptic characteristics, is provided in Figures S20–S21.

The detailed image contrast enhancement process is illustrated in Figure 5c. In this process, the enhancement of image contrast can be attributed to the dynamic modulation of the transfer characteristics in OSMTs. Conventional IGZO‐based phototransistors typically exhibit a relatively limited negative shift in their transfer curves, resulting in image arrays predominantly characterized by dark‐blue tones and consequently low contrast (Figure S22). On the contrary, by employing optical stimuli, OSMTs enable a substantially larger shift in the transfer characteristics compared to both conventional phototransistors and purely electronic memtransistors. This pronounced modulation facilitates conductance tunability, thereby leading to significantly enhanced image contrast. Here, the top and the bottom panel of the figure depict the operation based on electrical and optical stimuli, respectively. For image processing, we started by reading the initial LRS current of 36 discrete devices, mapping the current values into an image. To further generate an image of capital letter “H,” 20 devices were subjected to either electrical (40 V, 200 ms) or optical (44.9 mW∙cm^−2^, 20 ms) write pulses. While both approaches successfully produced recognizable images of blue letter “H,” electrically written devices exhibited low contrast, whereas optically written devices yielded a much clearer image due to higher currents achieved at O‐LRS. Nevertheless, the image contrast remained suboptimal since the current ratio of O‐LRS to E‐LRS is not sufficiently high. Thus, to enhance the image contrast, a global erase operation was performed by applying negatively biased pulses (−30 V, 200 ms with fixed V_G_ of 5 V) to all 36 devices, followed by selective reprogramming of the 20 target pixels. The residual effect of the initial write pulses ensured that these pixels were not totally transitioned to E‐HRS, and subsequent targeted write pulses further enhanced the contrast, resulting in a sharper and more distinguishable image. The enhanced contrast enables clearer recognition of the letter “H,” analogous to the way the human visual system can reinterpret ambiguous stimuli after retinal processing. The evolution of the pixel current values in the optically stimulated OSMT array is quantitatively presented in Figure 5d–e. Figure 5d shows the current values of the array after the first write process. The dark blue bars represent the current values of the 20 pixel locations corresponding to the letter “H” whereas the light blue bars show the current values of the remaining 16 pixels. After the final write process, the dark blue bars reach up to 10^−7^ A while the light blue bars decrease down to 10^−11^ A, evidently showing a significant enhancement of image contrast (Figure 5e). The evolution of pixel current values of the electrically processed OSMT array are also demonstrated in Figure S23. Figure 5f demonstrates the contrast ratio obtained from the initial and final images. Here, the contrast ratio is defined as the average current of 20 pixels divided by the average current of 16 background pixels. Additionally, “EW” and “OW” designate electrical‐write and optical‐write operations, corresponding to state modulation induced by electrical bias application and optical excitation, respectively. In the case of electrical image processing, the contrast ratio increased from 3.63 to 44.06 after image processing. An even higher increase in contrast ratio was observed during optical image processing, where the ratio increased by ∼520 times (7.46 to 3.88 × 10^3^), demonstrating the image processing capability of OSMTs.

To ensure the practicability of OSMT‐based retinomorphic visual processing system, we performed time‐resolved photoresponse measurements utilizing a high‐speed characterization framework to unveil the intrinsic temporal dynamics of OSMTs (Figure S24). We extracted rise time(*τ*_rise_) and fall time(*τ*_fall_) of OSMTs, where *τ*_rise_ is defined as the duration required for the current to increase from 10% to 90% of its peak value, whereas *τ*_fall_ corresponds to the time taken for the current to decay from 90% to 10% of the maximum current. The intrinsic *τ*_rise_ and *τ*_fall_ were quantified as 9.33 ms and 52.67 ms, respectively, demonstrating the device's capability to respond to realistic visual stimulus.

Furthermore, under standard operational conditions, the PPC characteristics of the IGZO channel induce a cumulative background current when subjected to continuous high‐frequency pulse trains, which obscures the discrete peak‐to‐peak amplitude necessary for a mathematically rigorous 3 dB bandwidth extraction. Although alternative characterization strategies such as applying a continuous large negative gate‐bias (−20 V) to suppress the PPC current could be used, they introduced an electro‐optical trade‐off where low‐frequency photoresponses were entirely annihilated (Figure S25), thereby preventing the establishment of a continuous, full‐spectrum frequency attenuation. Nevertheless, testing under a stimulus frequency of 10 kHz confirmed that the cumulative photoresponse still scales proportionally with the number of applied optical pulses. This indicates that the IGZO channel successfully perceives and integrates high‐frequency inputs.

Finally, to analyze the energy consumption during image processing, an array‐level demonstration was performed using the OSMTs having a channel length of 20 µm, which exhibited lowered operating voltages as shown in Figure S3. When comparing the operating pulse parameters at the single‐device level, the read current achieved by the 20 µm channel length device under a 10 V write pulse (∼ 400 pA) was comparable to that of 40 µm channel length device under a 40 V write pulse (ranging from 400 to 600 pA), confirming that similar conductance modulation can be achieved at a lower voltage range (Figure S26). Based on these results, the cumulative energy consumption of the array during a single writing process of the image processing task was evaluated. The 20 µm channel length OSMT array required a normalized energy of ∼20.56 nJ, showcasing a prominent enhancement in energy efficiency compared to the 41.44 nJ consumed by the 40 µm channel length OSMT array (Figure S27). These explicitly confirm that modulating device structural parameters to lower the operating voltage holds potential to facilitate the realization of energy‐efficient and linear hardware synapses, establishing a framework for future neuromorphic computing hardware.

## 3 Conclusion

In conclusion, we demonstrated a neuromorphic vision system for image processing by widely tuning the conductance states from E‐HRS to O‐LRS, offering the potential to be integrated into next‐generation neuromorphic vision applications. The resistive states of the device arise from the combination of a photoresponsive IGZO channel and field‐induced migration of oxygen vacancies within the asymmetrically positioned HfO_2_ interlayer. These states can be dynamically modulated by controlling the optoelectronic inputs, thereby enabling extensive range of conductance control. Moreover, key aspects of biological synaptic plasticity were mimicked via both optical and electrical stimuli. Through the emulation of synaptic plasticity, we achieved high recognition accuracy in ANN simulations, showcasing high applicability of OSMTs for optoelectronic neuromorphic computing. Most importantly, a 6 × 6 OSMT array was fabricated to present an image processing system. Each OSMT device serves the role as a biological retina and an electrical synapse simultaneously, directly responding to photonic input and modulating electrical synaptic weight for image processing. We believe these results establish OSMTs as highly promising building blocks for advanced neuromorphic vision systems and provide a strong foundation for the development of intelligent vision technologies that truly replicate the outstanding features of the biological vision system.

## 4 Experimental Section

### 4.1 Fabrication of OSMTs

A heavily doped p‐type Si substrate with an oxidized 200 nm‐thick SiO_2_ layer was used for the fabrication of OSMTs, where the p‐Si wafer and SiO_2_ layer were employed as the gate electrode and the gate insulator, respectively. A 10 nm‐thick IGZO active channel layer was deposited onto the gate insulator by RF magnetron sputtering at room temperature. Post‐deposition annealing of the IGZO layer was carried out at 400°C for 1 h in air ambient, followed by patterning via photolithography and wet etching. Subsequently, a 7.5 nm‐thick HfO_2_ interlayer was again deposited by RF magnetron sputtering and patterned by a lift‐off process. Here, the HfO_2_ interlayer was only formed at the channel/drain contact region, resulting in an asymmetric‐contact structure. This step was followed by an additional thermal annealing process at 300°C for 1 h in air ambient. Finally, 30 nm‐thick Al source/drain (S/D) electrodes were deposited by thermal evaporation and patterned via a lift‐off process. The resulting OSMTs had channel dimensions of 40 µm in length and 200 µm in width.

### 4.2 Optoelectronic Measurements

The optoelectronic characteristics of OSMTs were measured with semiconductor parameter analyzers (Agilent Technologies, 4155C, 4156C; Keithley, 4200A‐SCS; and Keysight, B1500A) in dark ambient condition. For applying optical stimuli, UV light‐emitting diode (peak wavelength of 405 nm) was utilized. For the emulation of potentiation and depression behaviors, arbitrary function generators were used (Tektronix, AFG 3022 and 31022).

### 4.3 Simulation of MNIST Digit Pattern Recognition

The NeuroSim+ platform was employed for a pattern recognition test to evaluate the applicability of OSMTs for neuromorphic computing [43]. For the training process, the adaptive moment estimation weight update method was used. For actual recognition tests, MNIST dataset was utilized.

### 4.4 Simulation of CIFAR‐10 Image Recognition

The DNN+NeuroSim framework was employed for the CIFAR‐10 recognition test to evaluate the applicability of OSMTs for complex visual processing [42]. The experimentally obtained optoelectronic LTP/LTD characteristics were incorporated into the synaptic weights of the CNN model for performance evaluation. The CIFAR‐10 dataset consisting of high‐dimensional color images was utilized for actual recognition tests.

## Author Contributions

**Donghyun Kang**: Conceptualization, Data curation, Formal analysis, Investigation, Methodology, Software, Writing – original draft, Validation. **San Nam**: Conceptualization, Data curation, Formal analysis, Investigation, Methodology, Software, Writing – original draft. **Dayul Nam**: Formal analysis, Investigation, Methodology. **In‐Soo Kim**: Formal analysis, Methodology. **Myung‐Gil Kim**: Formal analysis, Methodology. **Sung Kyu Park**: Conceptualization, Data curation, Formal analysis, Investigation, Methodology, Writing – original draft, Supervision. **Yong‐Hoon Kim**: Conceptualization, Data curation, Formal analysis, Funding acquisition, Investigation, Methodology, Resources, Supervision, Validation, Visualization, Writing – original draft.

## Conflicts of Interest

The authors declare no conflicts of interest.

## Supporting information




**Supporting File**: advs76218‐sup‐0001‐SuppMat.docx.

## Data Availability

The data that support the findings of this study are available from the corresponding author upon reasonable request.
