# Supplementary material for: Retinomorphic Visual Processing Enabled by Contact‐Engineered IGZO Optoelectronic Synaptic Memtransistors
Source: Adv Sci (Weinh). 2026 Jun 25:e76218. Online ahead of print. doi: 10.1002/advs.76218 (PMC13337062; doi:10.1002/advs.76218)
Supplement: Supplementary file 1 — Supporting File: advs76218‐sup‐0001‐SuppMat.docx. [file ADVS-9999-e76218-s001.docx]

Supporting Information

Retinomorphic Visual Processing Enabled by Contact-Engineered IGZO Optoelectronic Synaptic Memtransistors

Donghyun Kang, San Nam, Dayul Nam, In-Soo Kim, Myung-Gil Kim, Sung Kyu Park^*^, and Yong-Hoon Kim^*^

Donghyun Kang, San Nam, Prof. Myung-Gil Kim, Prof. Yong-Hoon Kim

School of Advanced Materials Science and Engineering, Sungkyunkwan University, Suwon 16419, Republic of Korea

E-mail: yhkim76@skku.edu

In-Soo Kim

Department of Medicine, University of Connecticut Health Center, Farmington, CT 06030, USA

Dayul Nam, Prof. Sung Kyu Park

School of Electrical and Electronics Engineering, Chung-Ang University, Seoul 06974, Republic of Korea

E-mail: skpark@cau.ac.kr

D. K. and S. N. contributed equally to this work.

***Corresponding authors**: Prof. Yong-Hoon Kim ([yhkim76@skku.edu](mailto:yhkim76@skku.edu)), and Prof. Sung Kyu Park ([skpark@cau.ac.kr](mailto:skpark@cau.ac.kr))

**This PDF file includes:**

Supporting Figures 1 to 27


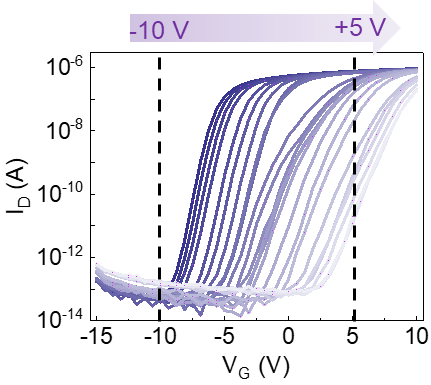


**Figure S1.** Evolution of transfer characteristics from O-LRS to E-HRS. The initial O-LRS was programmed by UV pulse, which was followed by stepwise negative V_D_ pulses (-40 V) with increasing pulse widths to gradually switch the device into E-HRS.

**
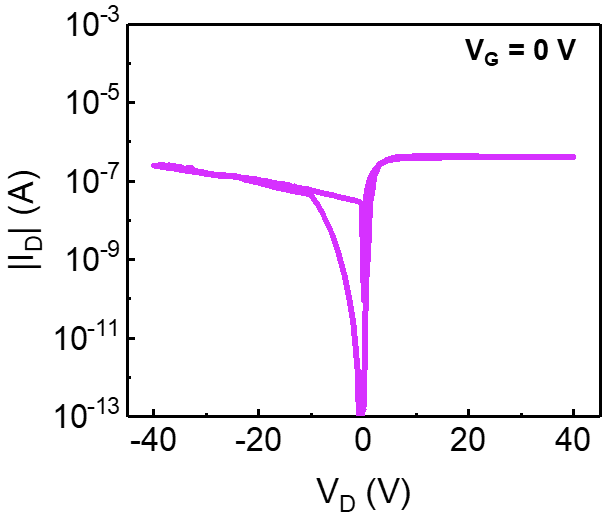
**

**Figure S2.** The resistive switching characteristics (I_D_-V_D_) of optoelectronic synaptic memtransistors (OSMTs) under ultraviolet (UV) illumination. UV light (intensity = 29.9 mW∙cm^-2^) was continuously exposed during the positive V_D_ sweep, showing increased current level compared to that of electrically operated device. The resistive state was transitioned to HRS during the subsequent negative V_D_ sweep.


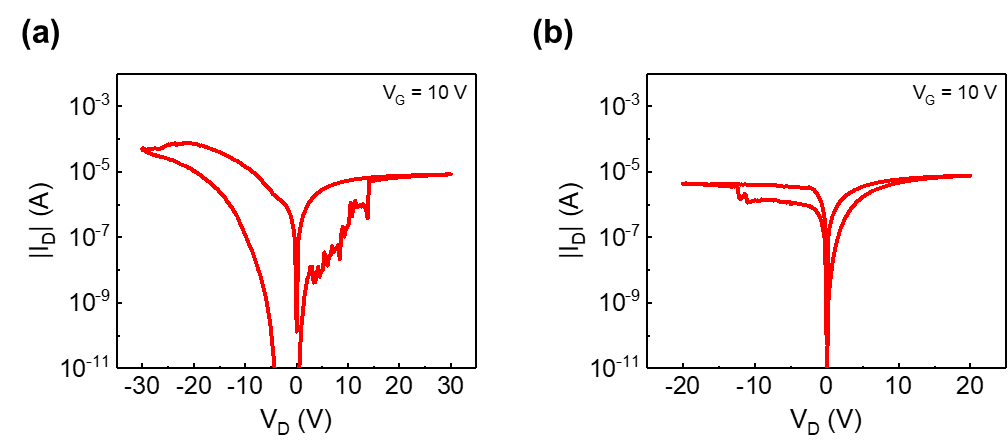


**Figure S3.** Resistive switching characteristics of HfO_2_ interlayer-based OSMTs having a channel length of 20 μm in the sweep range of (a) -30 V to 30 V, and (b) -20 V to 20 V.


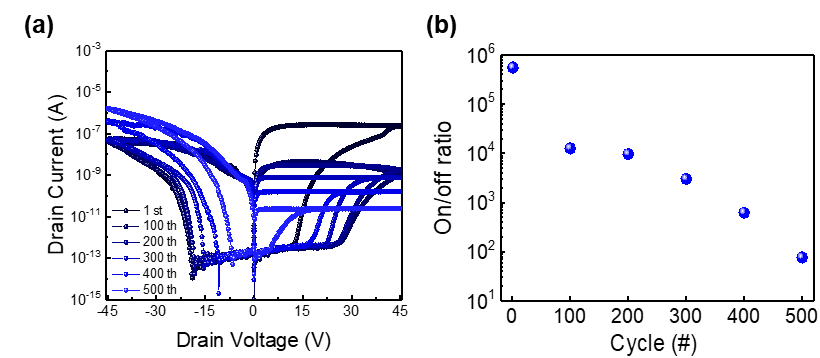


**Figure S4.** Extended endurance measurements with alternative measurement scheme (a) 500 repeated switching cycles at fixed V_G_ = 5 V with V_D_ sweep range of 45 V. (b) The on/off ratio measured at V_D_ = 5.175 V for each cycle number.


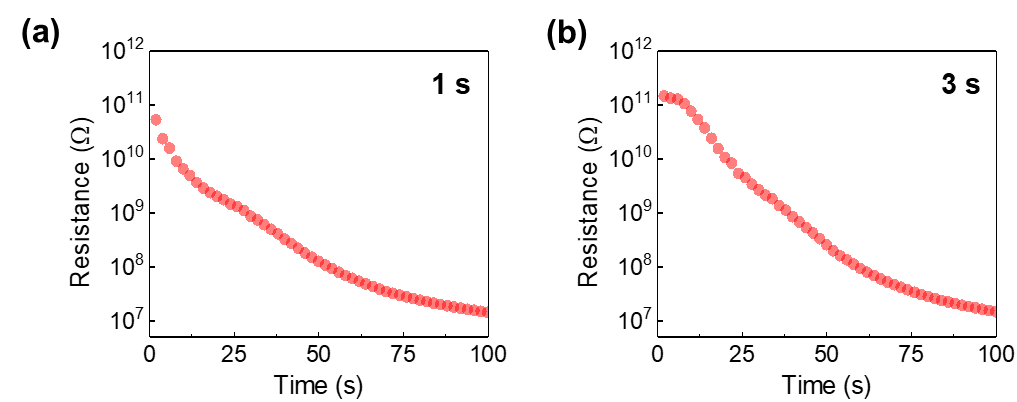


**Figure S5.** The short-term retention states of the OSMT in E-HRS triggered by varying negative electrical input duration, (a) 1 s, and (b) 3 s. As the input duration increases, the resistance decreases in a more gradual rate.

**
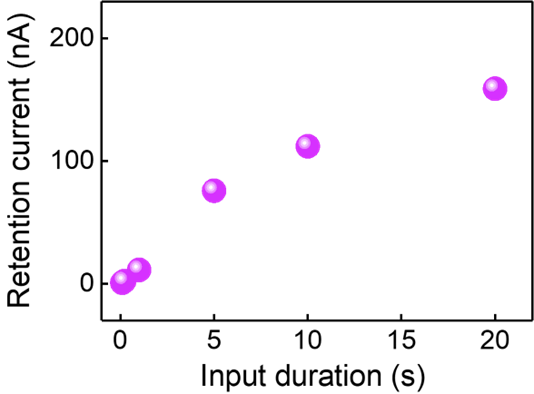
**

**Figure S6.** The variation in the retention current of OSMT as a function of optical input duration. Here, the retention current was defined as the current measured 30 s after the UV exposure. With shorter input durations, negligible retention current is attained, indicating short-term memory behaviour. On the other hand, longer input durations induce long-term memory behaviour, with the retention current reaching up to 160 nA.

**
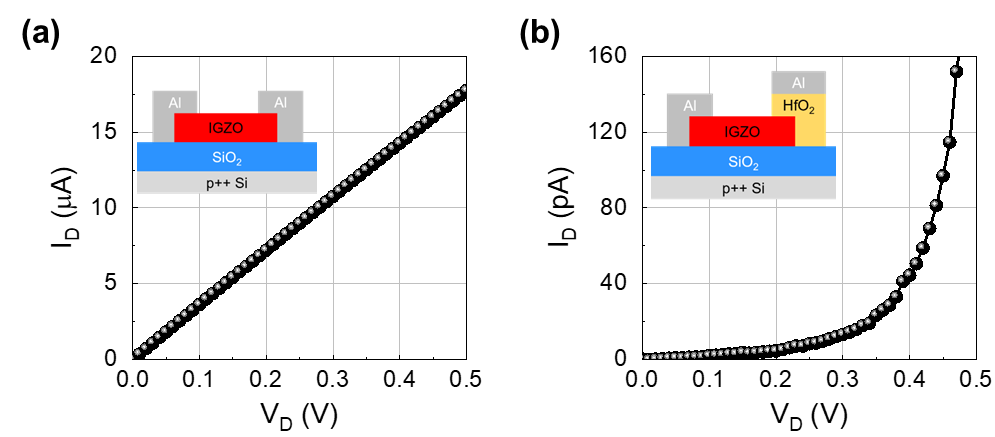
**

**Figure S7.** The output curves (I_D_-V_D_) of (a) indium-gallium-zinc-oxide (IGZO) transistor, and (b) OSMT plotted in a linear-scale. The linear curve of IGZO transistors indicate the formation of Ohmic contact whereas the nonlinear curve of OSMTs confirms the presence of Schottky contact at the drain/channel interface.

**
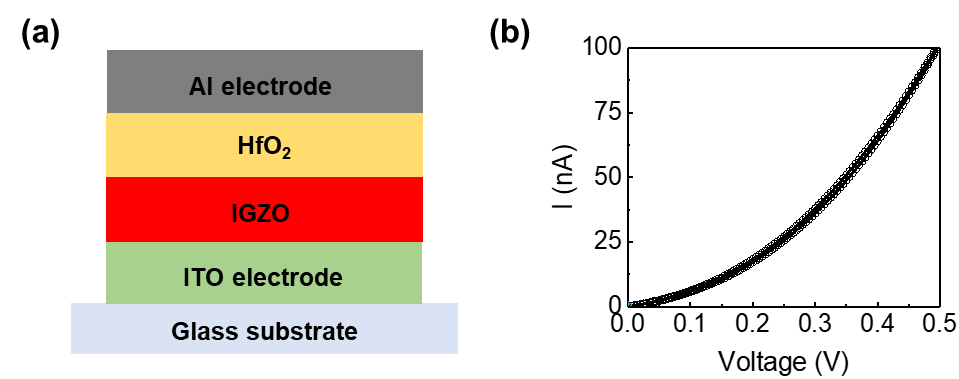
**

**Figure S8.** (a) Schematic of ITO/IGZO/HfO_2_/Al vertically stacked diode structure. Here, 10-nm thick IGZO and 7.5 nm-thick HfO_2_ layers were deposited on ITO bottom electrode sequentially. (b) I-V characteristic measured in the low voltage regime, demonstrating that the conductive property of HfO_2_ with Schottky contact characteristic.


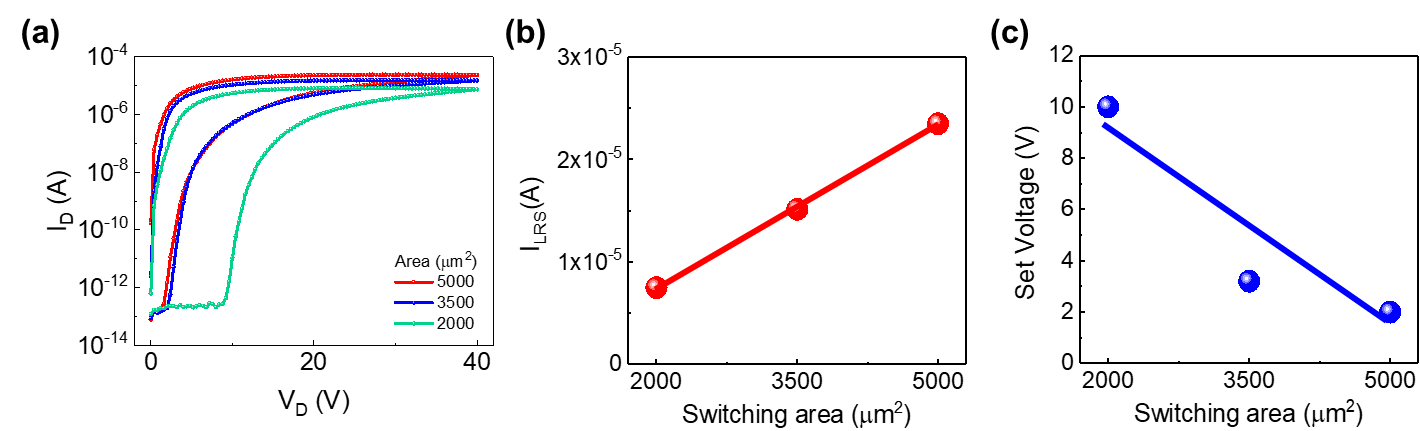


**Figure S9**. Area-dependent resistive switching properties of OSMTs. a) The I_D_-V_D_ curves of OSMTs measured at V_G_ = 15 V with varying switching interlayer area (2000, 3500, and 5000 μm^2^). The linear dependence between switching area and b) E-LRS current and c) Set voltage. Here, the set voltage is defined as the voltage point where the transition from E-HRS to E-LRS starts.


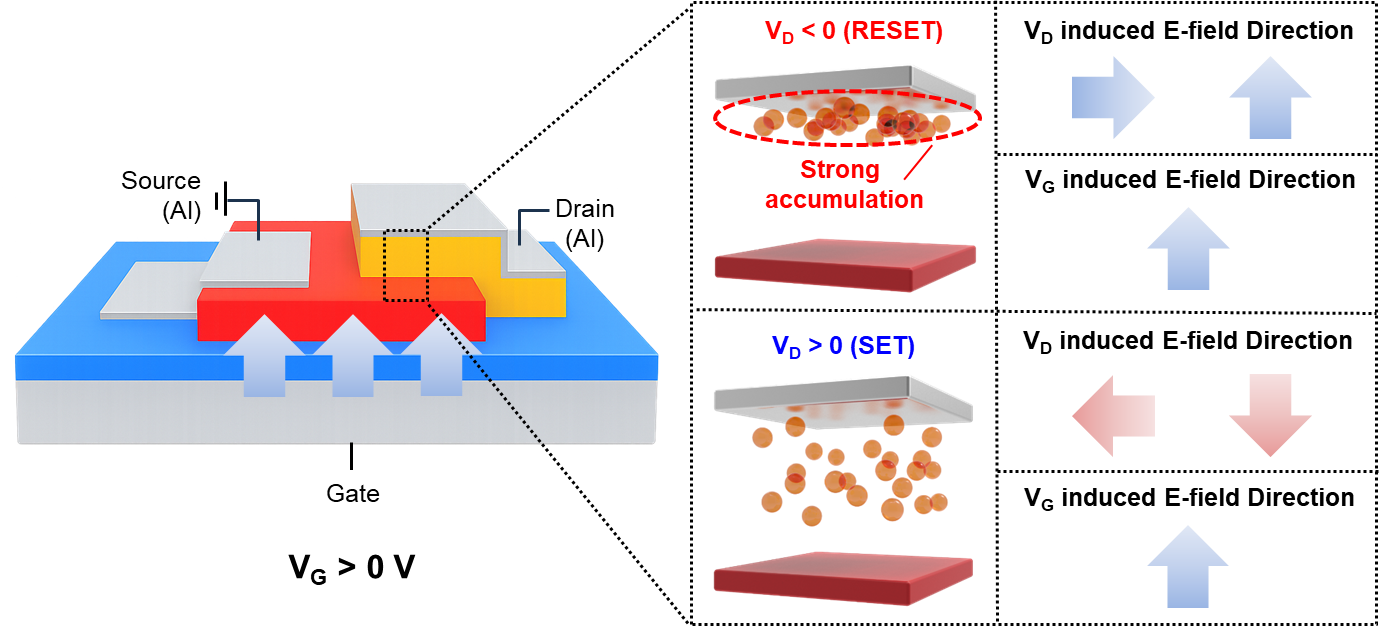


**Figure S10**. Electrical field-induced migration process of oxygen vacancies within the HfO_2_ interlayer. The arrows indicate the direction of the electric field. During both set and reset processes, a positive gate-bias is continuously applied, creating a vertical electrical field. When a negative drain bias is applied, both lateral and vertical electrical field are expected to be created. In this case, the directions of drain- and gate-induced field are identical. With the combined effect of two electric fields, the oxygen vacancies are strongly accumulated at the HfO_2_/drain interface, resulting in the rupture of conduction pathways. On the other hand, upon applying positive drain-bias, the directions of drain- and gate-induced electric field become opposite. Thus, we suppose that the oxygen vacancies drift toward HfO_2_/IGZO interface, but do not accumulate strongly enough to break the conduction pathways.

**
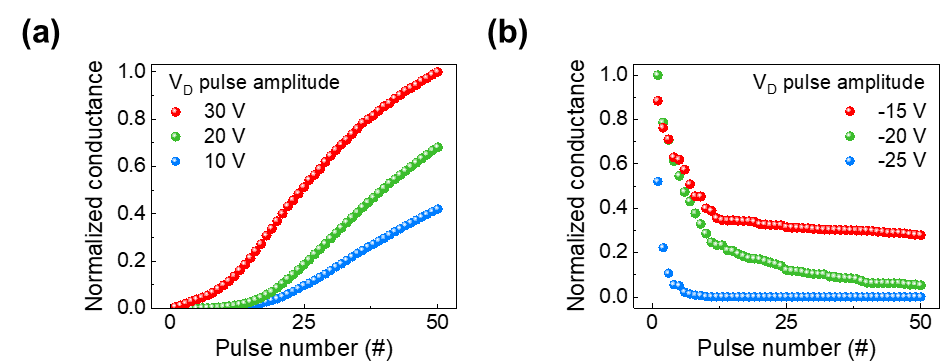
**

**Figure S11.** Voltage-dependent long-term potentiation (LTP) and long-term depression (LTD) behaviours of OSMT. 50 consecutive positive V_D_ pulses and negative V_D_ pulses with identical pulse width (200 ms) were implemented for the emulation of LTP and LTD, respectively. The results suggest that the device conductance exhibits a proportional dependence on the amplitude of the applied voltage pulses, leading to tunable LTP and LTD behaviours.


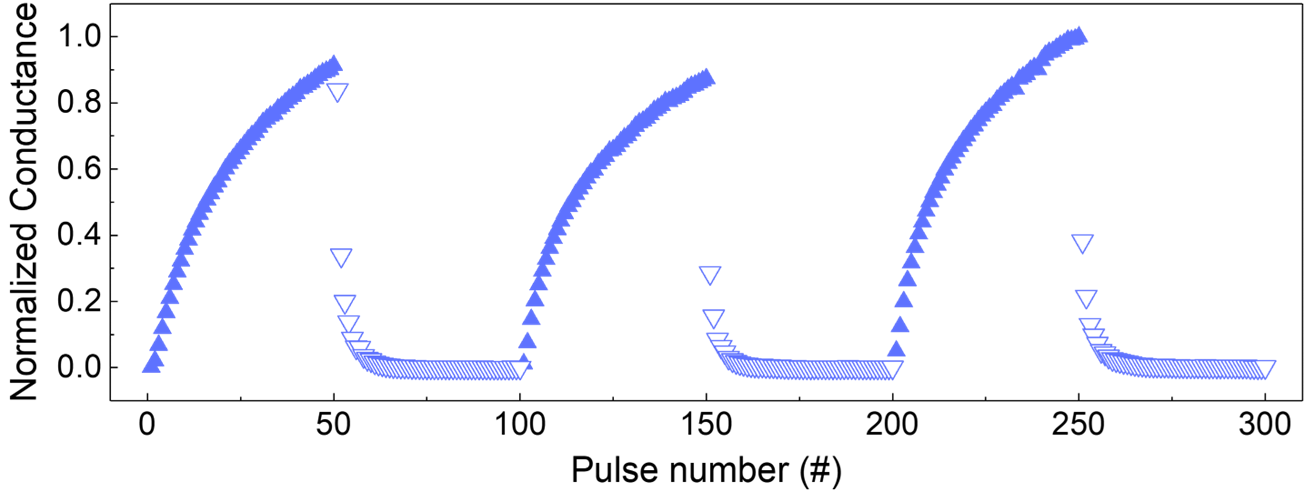


**Figure S12.** Endurance of potentiation and depression with positive and negative pulse train cycles. One cyclic pulse train consists of consecutive positive pulses (V_D_ = 30 V, V_G_ = 5 V, 50 pulses) followed by consecutive negative pulses (V_D_ = -25 V, V_G_ = 5 V, 50 pulses). This bidirectional pulse sequence was iterated three times to evaluate the reproducibility of synaptic functions.

**
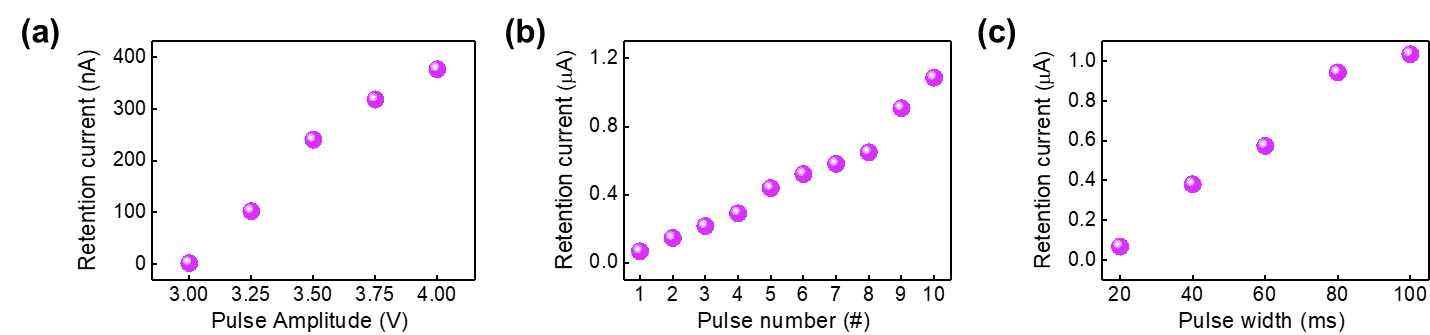
**

**Figure S13.** The change in retention current of optically stimulated OSMTs according to (a) pulse amplitude, (b) pulse number, and (c) pulse width. The increase in the retention current is proportional to the increase in the pulse amplitude, number, and width


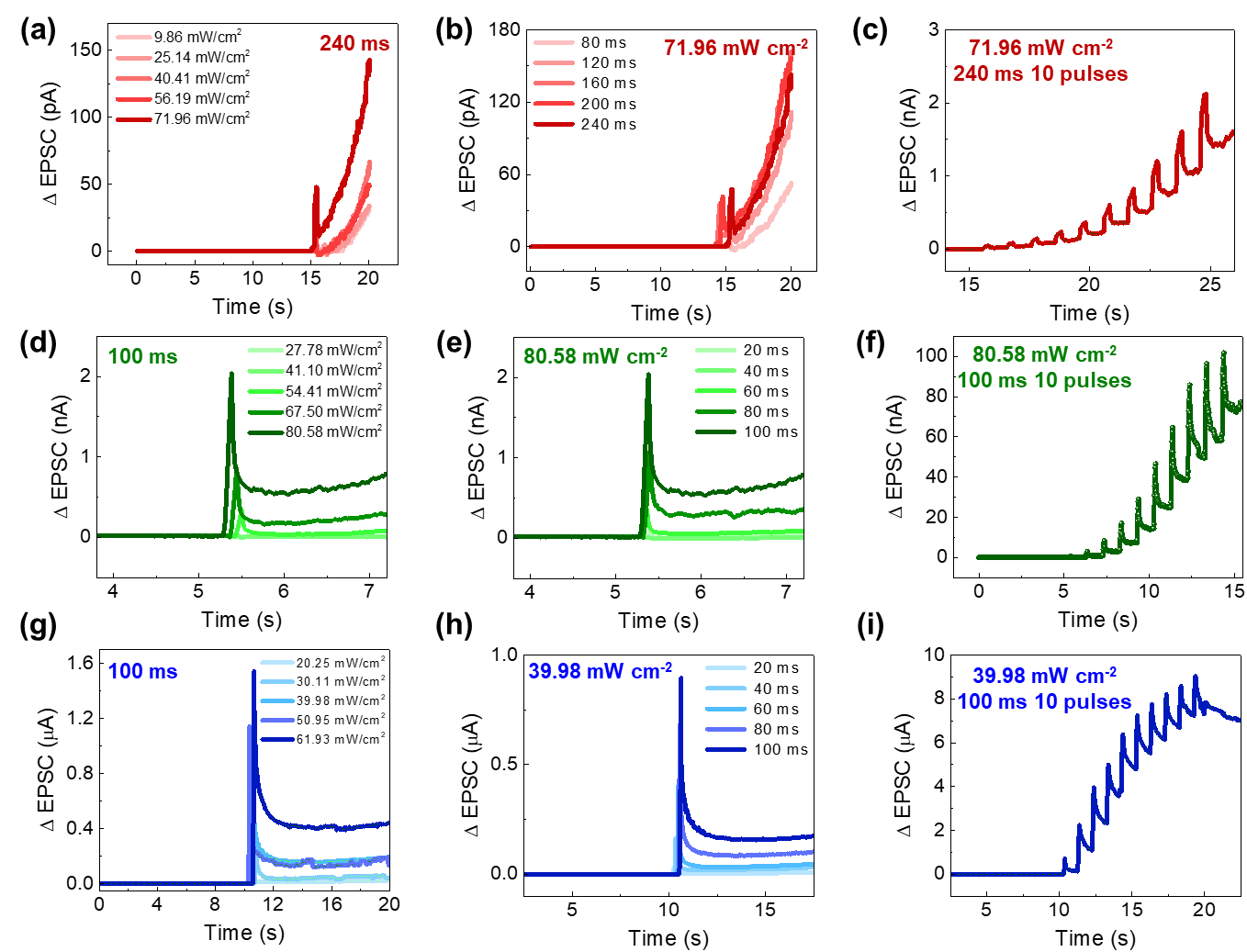


**Figure S14**. EPSC variation measured under various optical pulse conditions. EPSC variation as functions of a) pulse intensity and b) pulse width under red light illumination. c) Potentiation behavior emulated by 10 identical red-light pulses (71.96 mW∙cm^-2^, 240 ms). EPSC variation as functions of d) pulse intensity and e) pulse width under green light illumination. f) Potentiation behavior emulated by 10 identical green-light pulses (80.58 mW∙cm^-2^, 100 ms). EPSC variation as functions of g) pulse intensity and g) pulse width under blue light illumination. i) Potentiation behavior emulated by 10 identical blue-light pulses (39.98 mW∙cm^-2^, 100 ms).

**
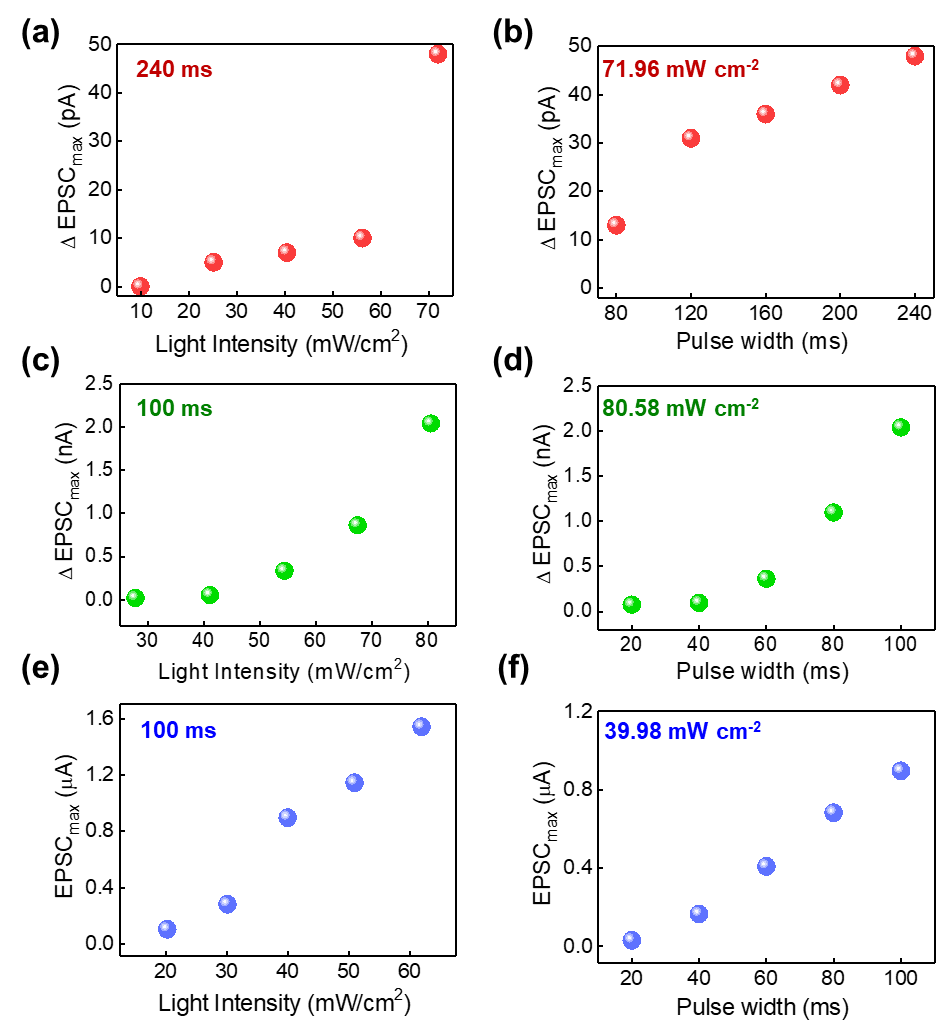
**

**Figure S15**. The variation of ΔEPSC as a function of light intensity measured with a) red-light, c) green-light, and e) blue-light. The variation of ΔEPSC as a function of pulse width measured with b) red-light, d) green-light, and f) blue-light.

**
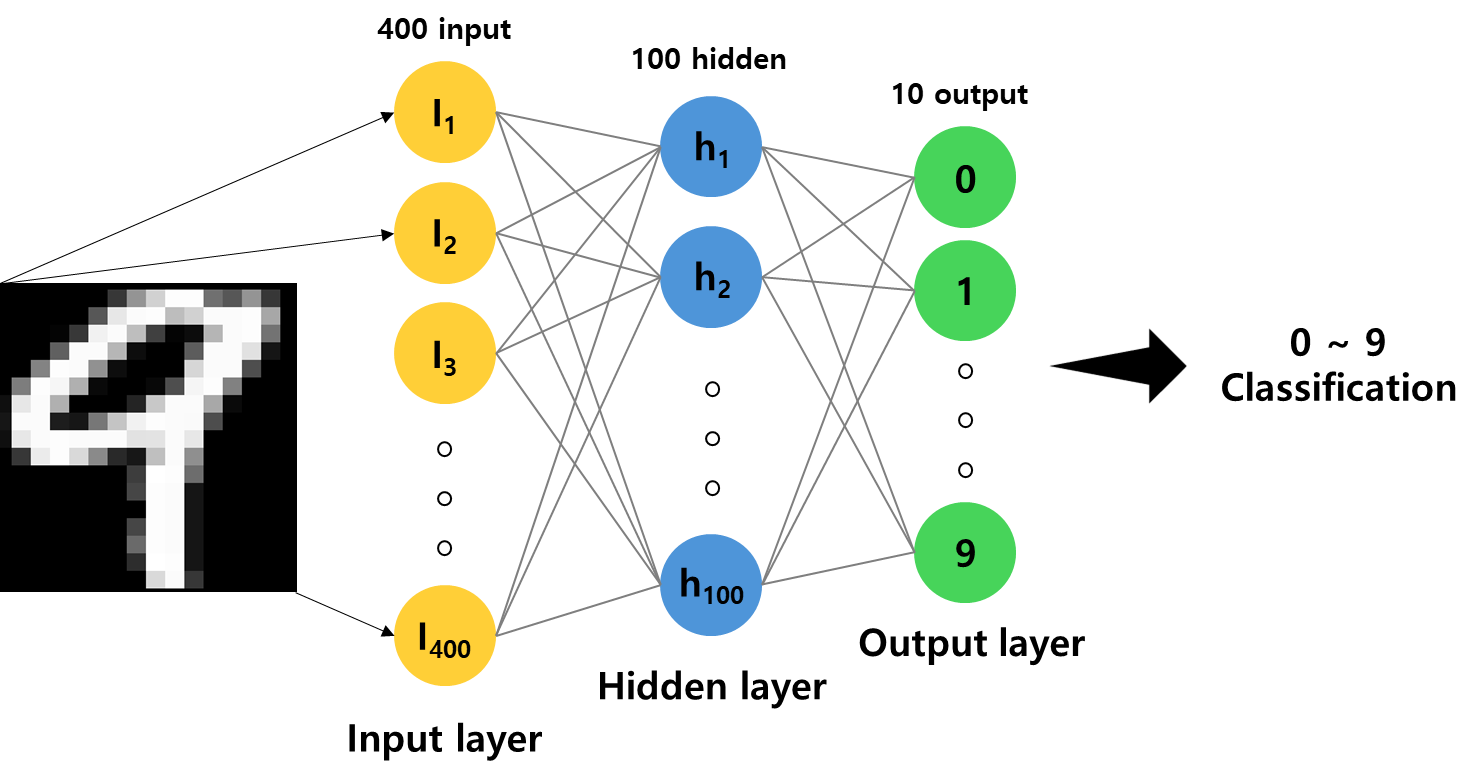
**

**Figure S16.** MNIST handwritten digit image and the multilayer perceptron-based artificial neural network scheme for digit classification.

**
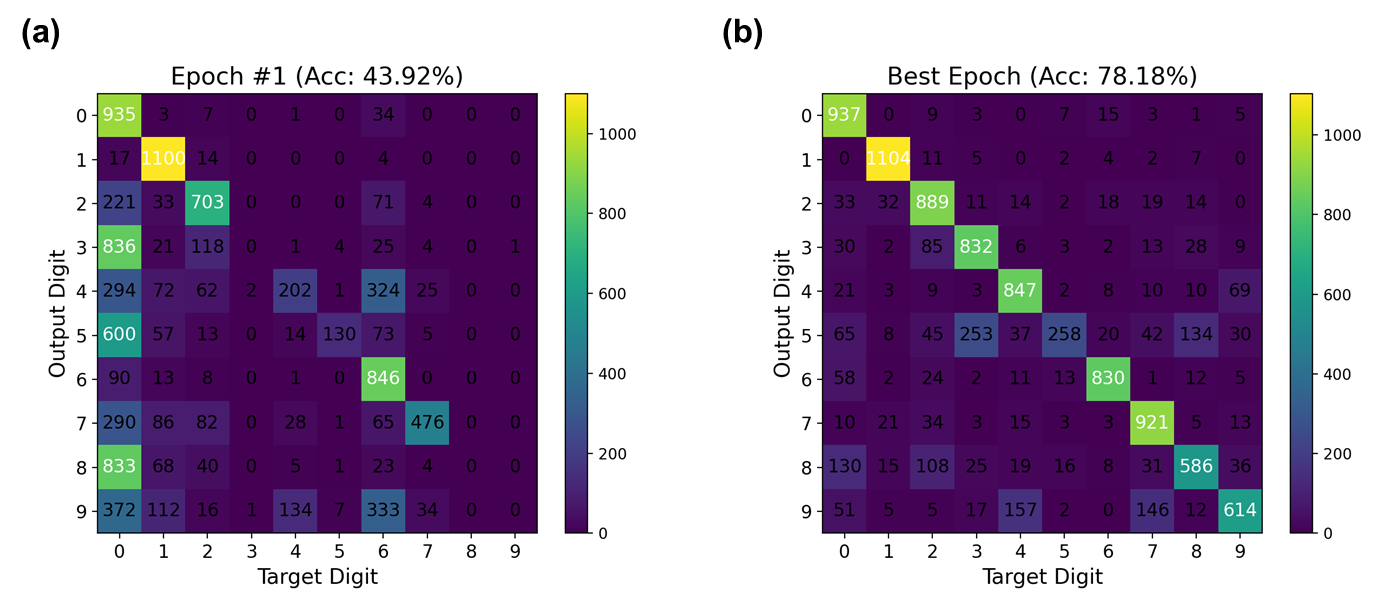
**

**Figure S17.** Confusion matrices corresponding to the epochs exhibiting the (a) lowest and (b) highest classification accuracies in the electrical potentiation/depression curves, respectively. The highest accuracy reached 78.18%, with moderate diagonal dominance.

**Figure S18**. Statistical analysis of the resistance state distribution in the 6x6 OSMT array. (a) LRS current distribution, (b) HRS current distribution, and (c) calculated LRS/HRS ratios for 36 individual devices measured at a fixed V_G_ of 10 V.

**Figure S19**. Raw data including the HRS current, LRS current, and calculated ratio for each of the 36 individual devices in the 6x6 OSMT array at a fixed V_G_ of 10 V.

**Figure S20**. Statistical analyses of maximum ΔEPSC for 36 OSMT devices.

**Figure S21**. Uniform and consistent LTP and LTD characteristics of 9 randomly selected devices in the 6x6 OSMT array

**
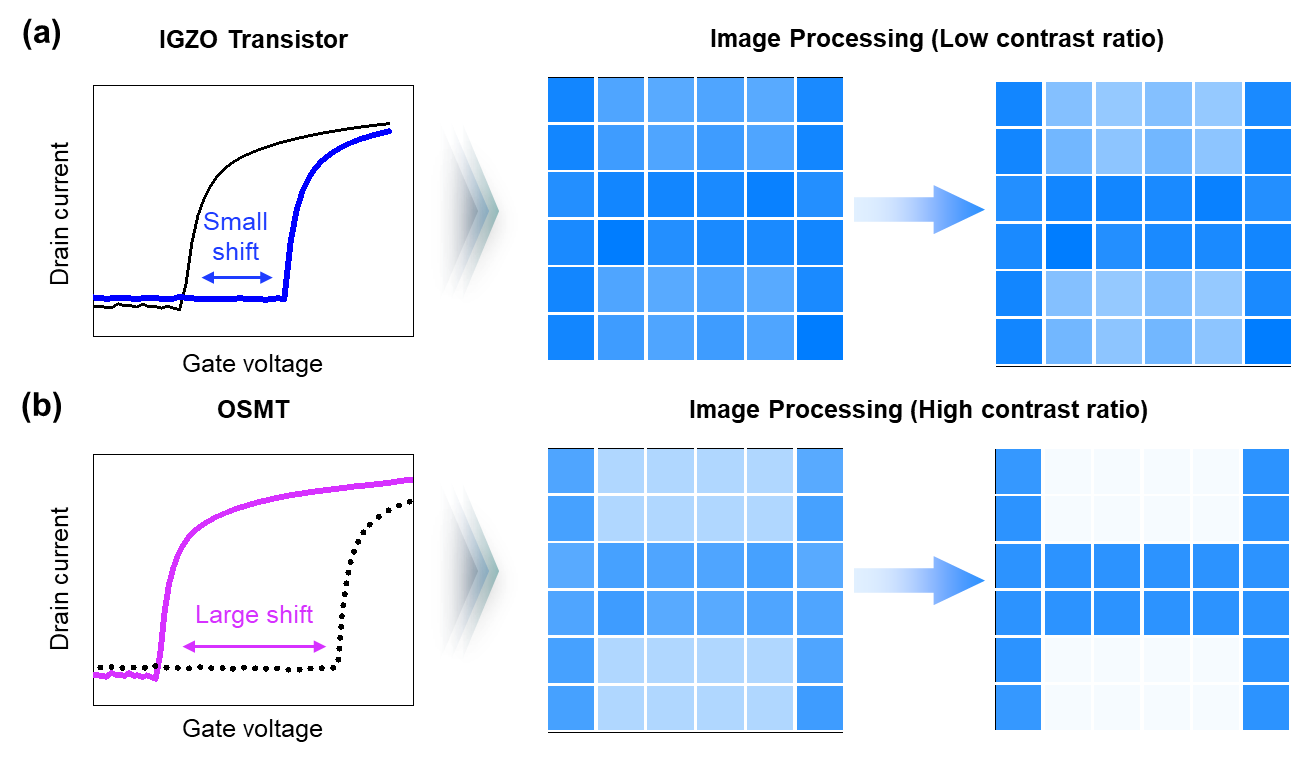
**

**Figure S22.** The mechanism for obtaining high contrast ratio during image processing. The left and right panel depict the shift in transfer characteristics and processed image array of (a) IGZO transistor and (b) OSMT, respectively. In typical IGZO phototransistors, the photo-induced shift in the transfer curve is relatively small, represented by the dark blue image array with low contrast. OSMTs however, exhibit larger range of shift in the transfer curve, thereby resluting in a high contrast image array with clear blue and white colors. It should be clarified that the current values presented in this figure are arbitrarily assigned for the purpose of conceptual explanation.

**
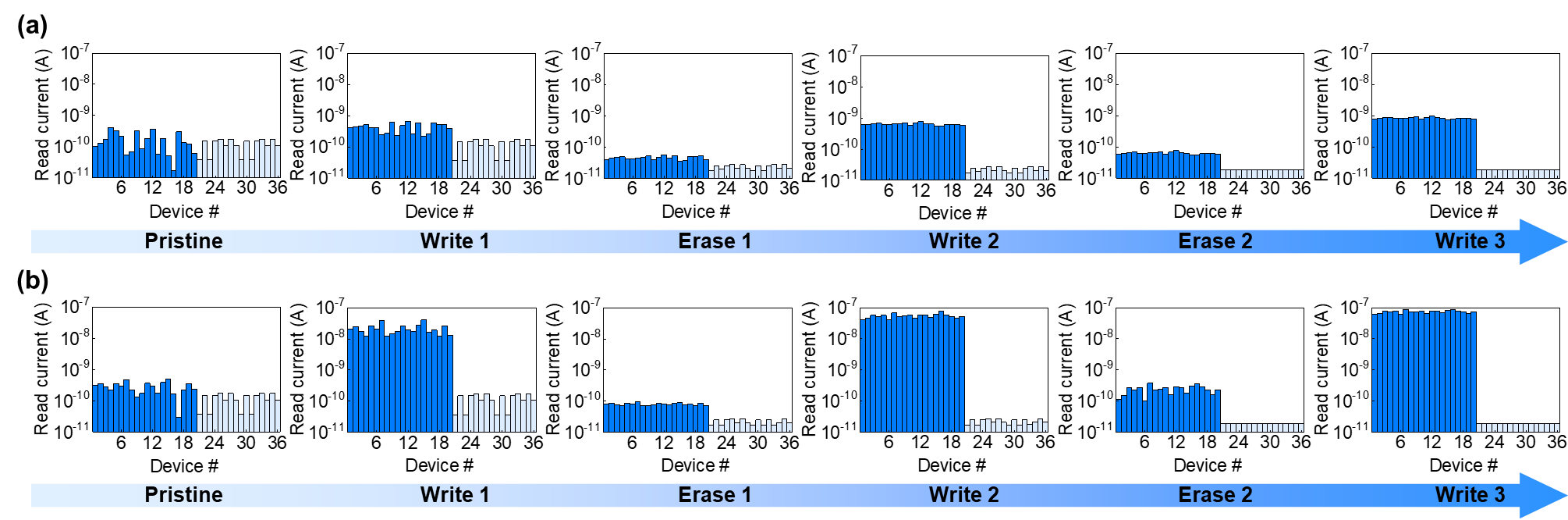
**

**Figure S23.** The evolution of pixel current values in (a) electrically and (b) optically processed OSMT array. Although the current ratio between targeted and background pixels progressively increases in both array configurations during image processing, the optically processed OSMT array exhibits a substantially more pronounced change in current value

**
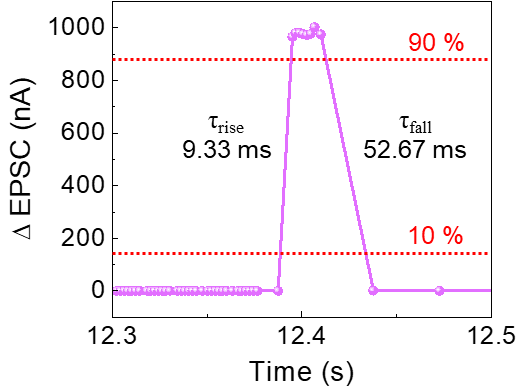
**

**Figure S24.** The extracted rise time (9.33 ms) and fall time (52.67 ms) of OSMT device under 10 ms UV pulse illumination.


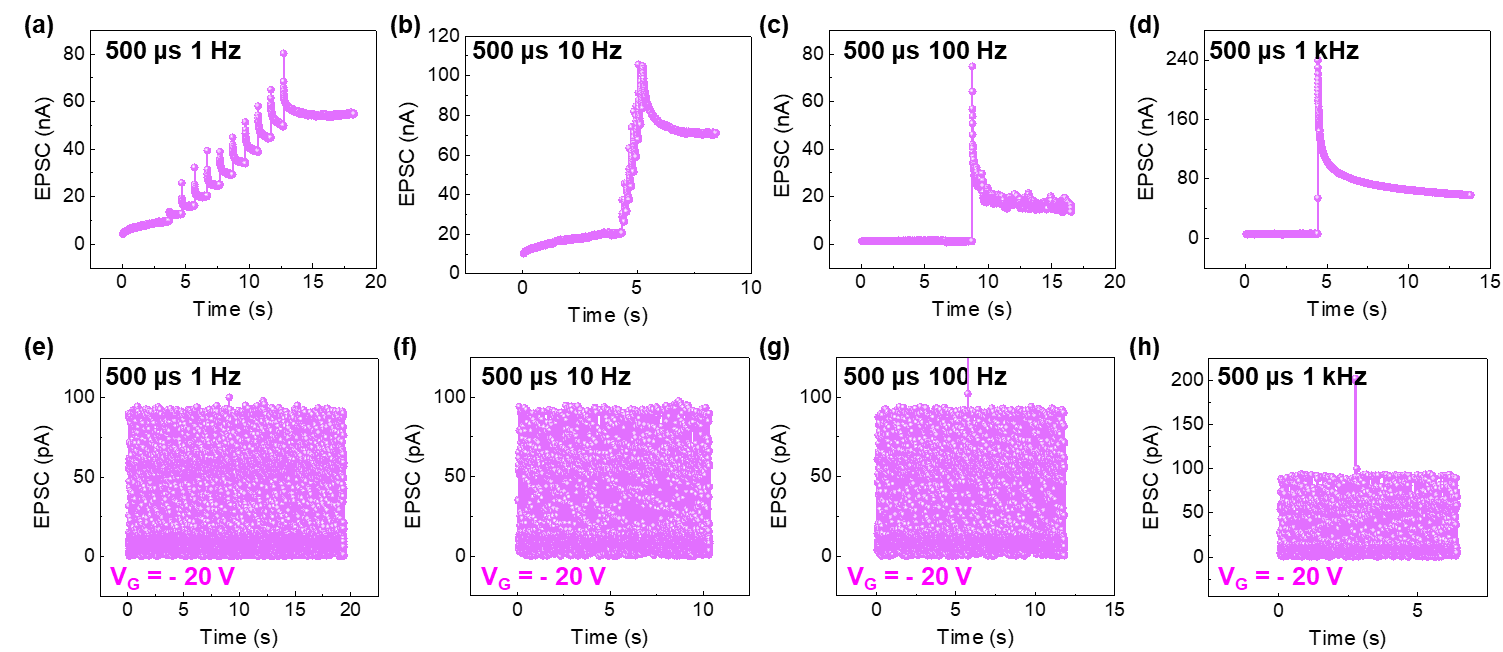


**Figure S25.** Frequency-dependent EPSC modulation of OSMTs. The frequency was varied as (a) 1 Hz, (b) 10 Hz, (c) 100 Hz, and (d) 1 kHz with fixed pulse width of 500 μs. To suppress the PPC, a negative gate-bias of -20 V was continuously applied during sampling with varying frequency of (e) 1 Hz, (f) 10 Hz, (g) 100 Hz, and (h) 1 kHz.


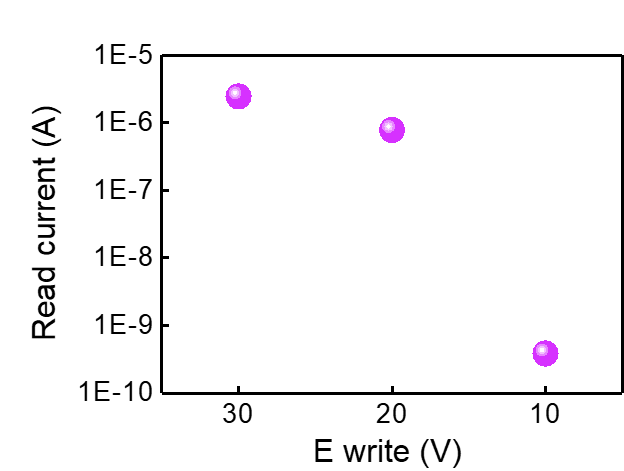


**Figure S26.** Potentiation pulses voltage modulation characteristics of the 20 μm channel length OSMT. The write voltage amplitude was varied from 30 V to 10 V in 10 V decrements under a fixed write pulse width of 200 ms and a gate bias of 10 V. The read operations were conducted under fixed conditions of V_G_ = 5 V and V_D_ = 3 V.


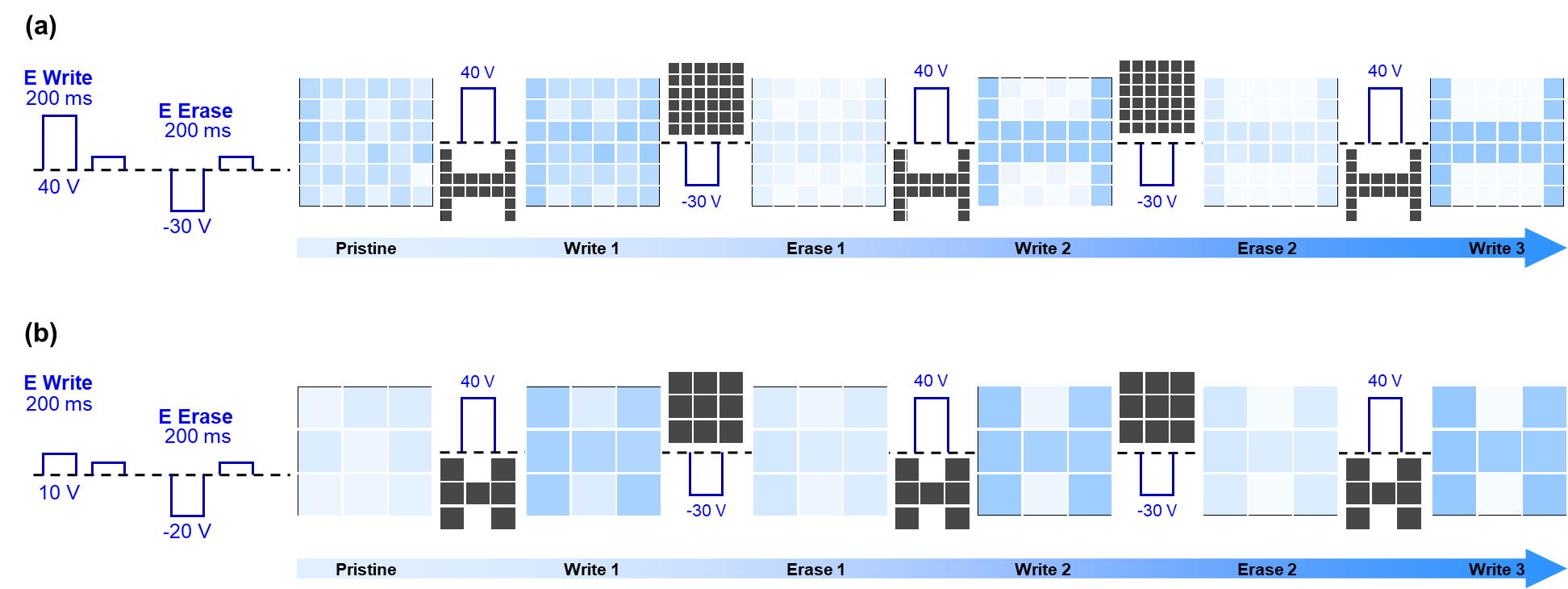


**Figure S27.** Demonstration of image processing task. (a) Image processing task utilizing a 6x6 OSMT array with a channel length of 40 μm. The write and erase pulses of identical width (200 ms) were utilized, with different amplitudes of 40 V and -30 V, respectively. (b) The 20 μm channel length 3×3 array during state transitions under a reduced pulse scheme. The write and erase pulses of identical width (200 ms) were utilized, with different amplitudes of 10 V and -20 V, respectively. The read pulses were globally fixed as V_G_ = 5 V and V_D_ = 3 V.
